# Distinct signalling dynamics of BMP4 and BMP9 in brown versus white adipocytes

**DOI:** 10.1038/s41598-025-99122-5

**Published:** 2025-05-08

**Authors:** Benjamin Constant, Ioannis Kamzolas, Xudong Yang, Jingxu Guo, Sonia Rodriguez-Fdez, Iman Mali, Sergio Rodriguez-Cuenca, Evangelia Petsalaki, Antonio Vidal-Puig, Wei Li

**Affiliations:** 1 Department of Medicine, VPD Heart and Lung Research Institute, School of Clinical Medicine, University of Cambridge, Papworth Road, Cambridge Biomedical Campus, Cambridge, CB2 0BB UK; 2 MRC Institute of Metabolic Science, MRC Metabolic Diseases Unit, Addenbrooke’s Hospital, University of Cambridge, Box 289, Cambridge, CB2 0QQ UK; 3 European Molecular Biology Laboratory, European Bioinformatics Institute (EMBL-EBI), Wellcome Genome Campus, Hinxton, Cambridge, UK; 4 CIBERDEN, Centro de Investigacion Principe Felipe, Valencia, Spain

**Keywords:** Extracellular signalling molecules, Morphogen signalling

## Abstract

Adipocyte dysfunction contributes to lipotoxicity and cardiometabolic diseases. Bone morphogenetic protein 4 (BMP4) is expressed in white adipocytes and remodels white adipose tissue, while liver-derived BMP9, a key circulating BMP, influences adipocyte lipid metabolism. The gene sets regulated by BMP4 and BMP9 signalling in mature adipocytes remain unclear. Here, we directly compare BMP4 and BMP9 signalling in mature brown and white adipocytes. While both BMPs showed comparable potency across adipocyte types, RNA sequencing analysis revealed extensive gene regulation, with many more differentially expressed genes and suppression of critical metabolic pathways in white adipocytes. Although BMP4 and BMP9 induced inhibitors of BMP and GDF signalling in both adipocytes, they selectively upregulated several TGF-β family receptors and BMP4 expression only in white adipocytes. These findings underscore a central role of BMP signalling in adipocyte homeostasis and suggest both BMP4 and BMP9 as regulators of white adipocyte plasticity with potential therapeutic implications.

## Introduction

The subcutaneous white adipose tissue (WAT), predominantly comprising white adipocytes, represents the largest fat depot in the human body and serves as a significant sink to accommodate excess energy storage^1^. Upon saturation of its storage capacity, lipids accumulate in non-adipose tissues, such as skeletal muscle, cardiac tissue, liver, and pancreas^1^, leading to lipotoxicity and a cascade of metabolic dysfunctions such as insulin resistance, beta cell dysfunction, hepatic steatosis and atherosclerosis^2,3^. In contrast, brown adipose tissue (BAT), located in smaller depots such as those around the neck and along major blood vessels^4^, mainly contains brown adipocytes. BAT can store lipids but preferentially metabolises them during oxidative phosphorylation to facilitate uncoupling protein 1 (Ucp1)-mediated thermogenesis. BAT can act as a sink to eliminate excess nutrients and prevent lipotoxicity. Enhanced BAT activity correlates with increased energy expenditure and potential obesity mitigation.

The adipose tissue is a highly plastic organ, having a unique ability to expand and shrink within the same individual over time and in response to various nutritional and hormonal changes^5^, due to alterations in the size and number of adipocytes^6,7^. Over 10% of stem and stromal cells are found in different adipose tissue depots^5^. Pre-adipocytes can be isolated from WAT and BAT and differentiated into mature white and brown adipocytes^8,9^. White adipocytes can convert to brown-like adipocytes under metabolic stress such as cold and fasting^10,11^.

Bone morphogenetic proteins (BMPs) are secreted growth factors regulating bone and cartilage formation, as well as organ development and homeostasis in various tissues^12^. They signal by forming a signalling complex with two copies of a type I receptor and two copies of a type II receptor, mainly through the Smad1/5-dependent, some also via Smad-independent pathways. BMPs regulate many processes in adipose tissues, including the formation of adipose tissue, the dynamic regulation of its plasticity and thermogenic programming^13^. Several BMPs play crucial roles in adipocyte differentiation. For example, BMP4 induces commitment of mesenchymal stem cells (MSCs) to adipocyte lineage but inhibits the acquisition of a brown phenotype during terminal differentiation^14^; BMP7 induces the brown adipocyte differentiation but has no effect on the white pre-adipocytes^15^, whereas BMP9 promotes brown adipocytes differentiation from pluripotent stem cells and stimulates browning of progenitor cells within WAT^16,17^. Regarding the regulation of adipose tissue plasticity, BMP4 was shown to regulate WAT remodelling and induction of brown adipocyte-like cell structure and function^8,18^.

Evidence suggests that BMP4 and BMP9 also play critical roles in vivo and in mature adipocytes. BMP4 is enriched in WAT and positively correlates with obesity^14^. BMP4 suppresses lipolysis via regulation of hormone-sensitive lipase expression, and there is a strong correlation between BMP4 levels and adipocyte size as well as insulin sensitivity in humans^14^. BMP9 was reported to play a significant role in glucose and lipid metabolism. Circulating BMP9 levels are markedly lower in essential hypertension patients^19^, in newly diagnosed type 2 diabetic patients^20^, as well as in patients with metabolic syndromes^21,22^. In preclinical models, BMP9 expression is reduced in insulin-resistant rats^23^; BMP9 administration reduced the size of white adipocytes and decreased 16-h fasting blood glucose levels without changes in food consumption^16^. In mice, BMP9 enhanced *Fgf21* expression, reduced the number of lipid droplets in the liver and alleviated many pathological symptoms of high-fat diet (HFD)-induced obesity^24^. BMP9-knockout mice exhibited hepatosteatosis due to down-regulated PPARα expression and reduced fatty acid oxidation^22^. Overexpression of hepatic BMP9 improved glucose tolerance and insulin resistance^25^, relieved liver steatosis and obesity-related metabolic syndrome^22,26^.

Despite the roles of BMP4 and BMP9 in lipid metabolism and adipose tissue function, the capability and the direct molecular outcome of BMP4 and BMP9 signalling in mature adipocytes have not been reported. It is unclear whether BMP4 and BMP9 signalling regulates similar target genes in brown and white adipocytes, or whether each BMP (BMP4 or BMP9) exerts distinct roles in the mature brown and white adipocytes. In this study, we examined BMP4 and BMP9 signalling in fully differentiated mature brown and white murine adipocyte cell models, assessing BMP signalling potency in dose-dependent assays and downstream targets through RNA sequencing. Our data revealed that BMP4 and BMP9 induced changes in substantially overlapping gene sets in both brown and white adipocytes. Both BMPs regulated many more genes in white adipocytes than in brown adipocytes and suppressed critical metabolic pathways in white adipocytes. Interestingly, both BMP4 and BMP9 signalling induced changes in gene expression of many extracellular regulators of TGF-β family signalling, highlighting the importance of balanced TGF-β family signalling in the plasticity and homeostasis of white adipocytes.

## Results

### BMP9 signals with similar potency to BMP4 in mature brown and white adipocytes

**Fig. 1:**
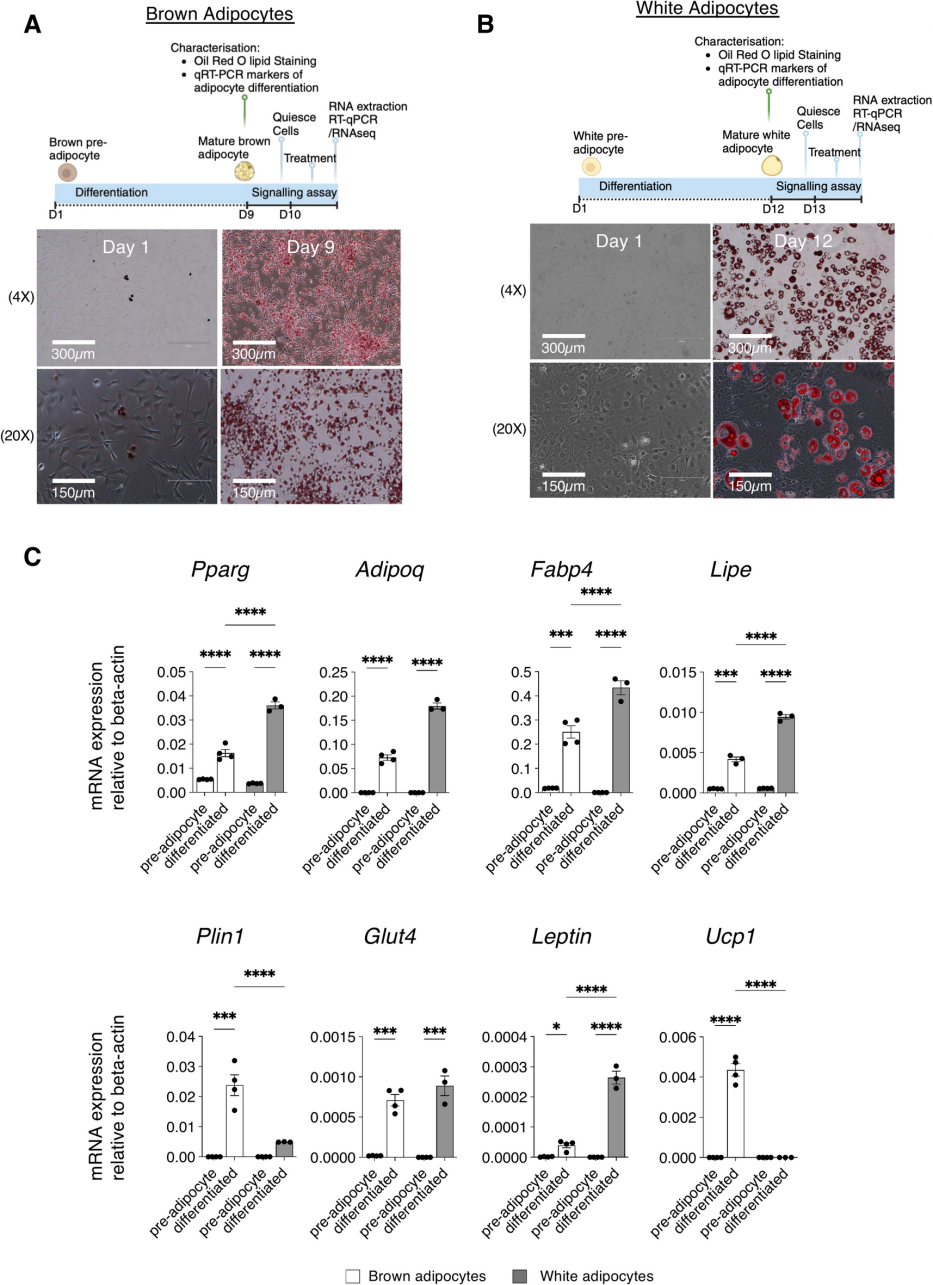
Characterisation of fully differentiated brown and white adipocytes. (**A**) Diagrams illustrating the differentiation of P-BAT brown pre-adipocytes (**A**) and 3T3-L1 white pre-adipocytes (**B**) and Oil red O staining of the cells before and after differentiation. Diagrams created in https://BioRender.com. (**C**) mRNA expression of mature adipocyte markers. N = 3 to 4, each datapoint is one independently differentiated sample, and mean ± SEM is shown. Two-way AVOVA mixed-effect model, *, *P* < 0.05, **, *P* < 0.01, ***, *P* < 0.001, ****, *P* < 0.0001.

Murine brown pre-adipocytes (P-BAT cells) and white pre-adipocytes (3T3-L1 cells) were differentiated into mature brown and white adipocytes (Fig. 1A,B), respectively, as characterised by positive Oil Red O staining (Fig. 1A,B). The expression of key adipocytes’ markers was induced after differentiation, including initiation factor *Pparg*, adipokine *Adipoq*, and genes involved in lipid transport, metabolism and storage (*Fabp4, Lipe* and *Plin1*), glucose uptake (*Glut4)* (Fig. 1C). *Leptin* was preferentially expressed in mature white adipocytes, whereas *Ucp1* was only expressed in the mature brown adipocytes (Fig. 1C).

**Fig. 2:**
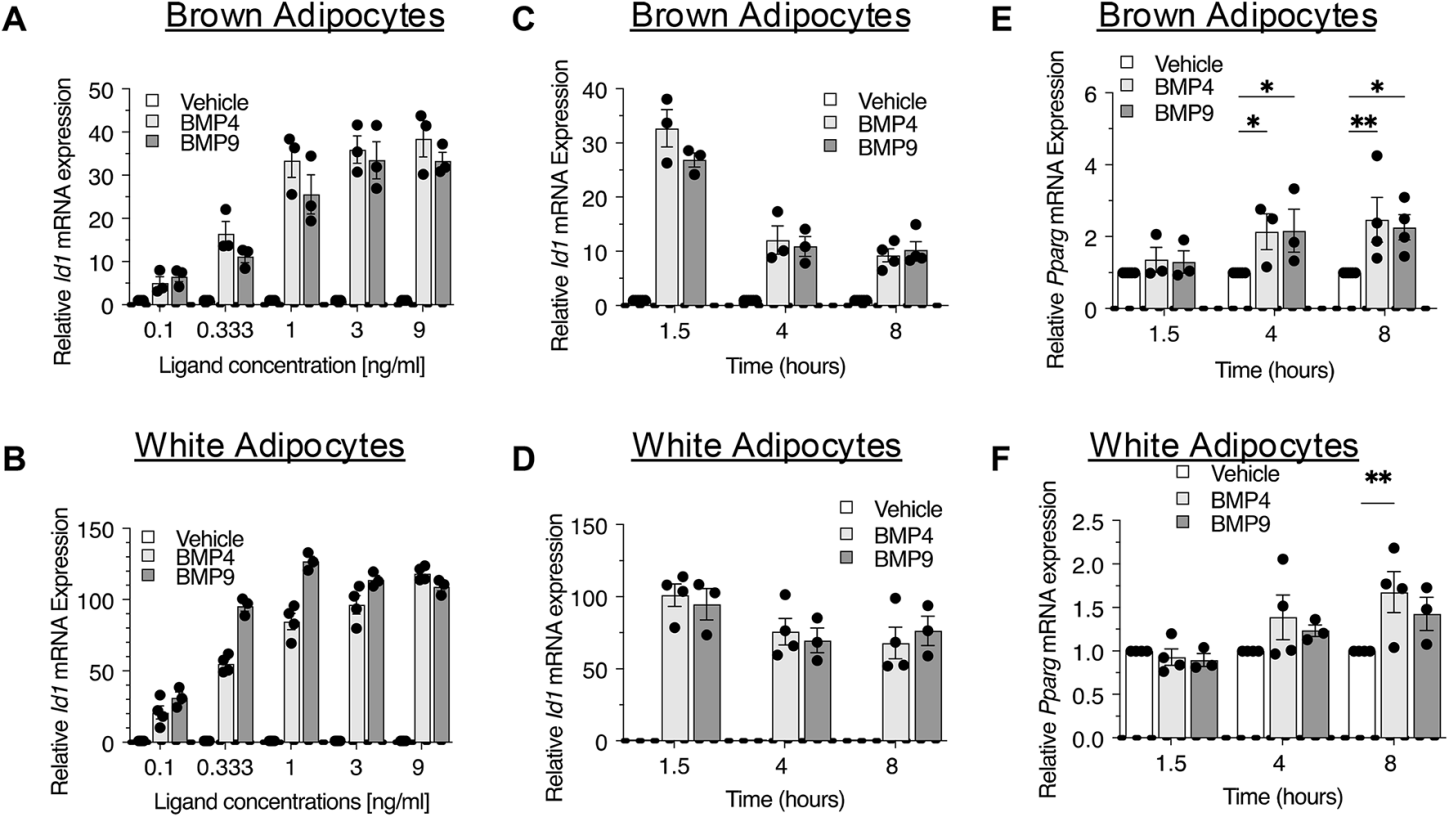
Comparison of BMP4 and BMP9 signalling in mature brown and white adipocytes by dose–response and time-course signalling assays. (**A** and **B**) Dose-dependent signalling in brown (**A**) and white (**B**) mature adipocytes. The treatment time is 1.5 h. (**C** and **D**) Time course signalling assays, monitored by *Id1* gene induction. (**E** and **F**) Induction of master regulator PPARγ gene by BMP4 and BMP9 treatment in brown (**E**) and white (**F**) adipocytes. Means ± SEM are shown, Data from 3 or 4 independently differentiated brown or white adipocytes. (**E** and **F**), two-way ANOVA, *, *P* < 0.05, **, *P* < 0.01.

BMP4 and BMP9 induced dose-dependent upregulation of *Id1* in both adipocyte types with similar potency (Fig. 2A,B). A concentration of 3 ng/ml was selected for the time course experiment as the *Id1* induction at this concentration approached the plateau of the maximum response for both BMPs, thus ensuring a robust BMP response while minimising potential off-target effects with higher concentrations. BMP4- and BMP9-induced SMAD1/5 phosphorylation persisted for 8 h in both adipocyte types (Supplementary Fig. 1A&B). We then compared the ability of BMP4 and BMP9 to induce different target genes at different timepoints. In both brown and white adipocytes, at 1.5, 4 and 8 h post-treatment, BMP4 and BMP9 induced robust *Id1* gene expression, and no difference could be detected between them (Fig. 2C,D).

PPARγ, a transcription factor and nuclear receptor, is considered a key regulator of adipogenesis and controls the transcription of numerous downstream adipogenic and lipogenic genes^27,28^, also vital for maintaining mature adipocyte functionality, overseeing the expression of essential metabolic genes and enzymes^28,29^. We evaluated PPARγ (*Pparg*) gene expression at 1.5, 4 and 8 h post BMP4 and BMP9 treatment. In brown adipocytes, BMP4 and BMP9 significantly increased *Pparg* expression at both 4 and 8 h (Fig. 2E). In white adipocytes, significant *Pparg* mRNA induction could only be detected at 8 h after BMP4 treatment (Fig. 2F).

### Overview of transcriptomes induced by BMP4 or BMP9 in mature brown and white adipocytes

**Fig. 3:**
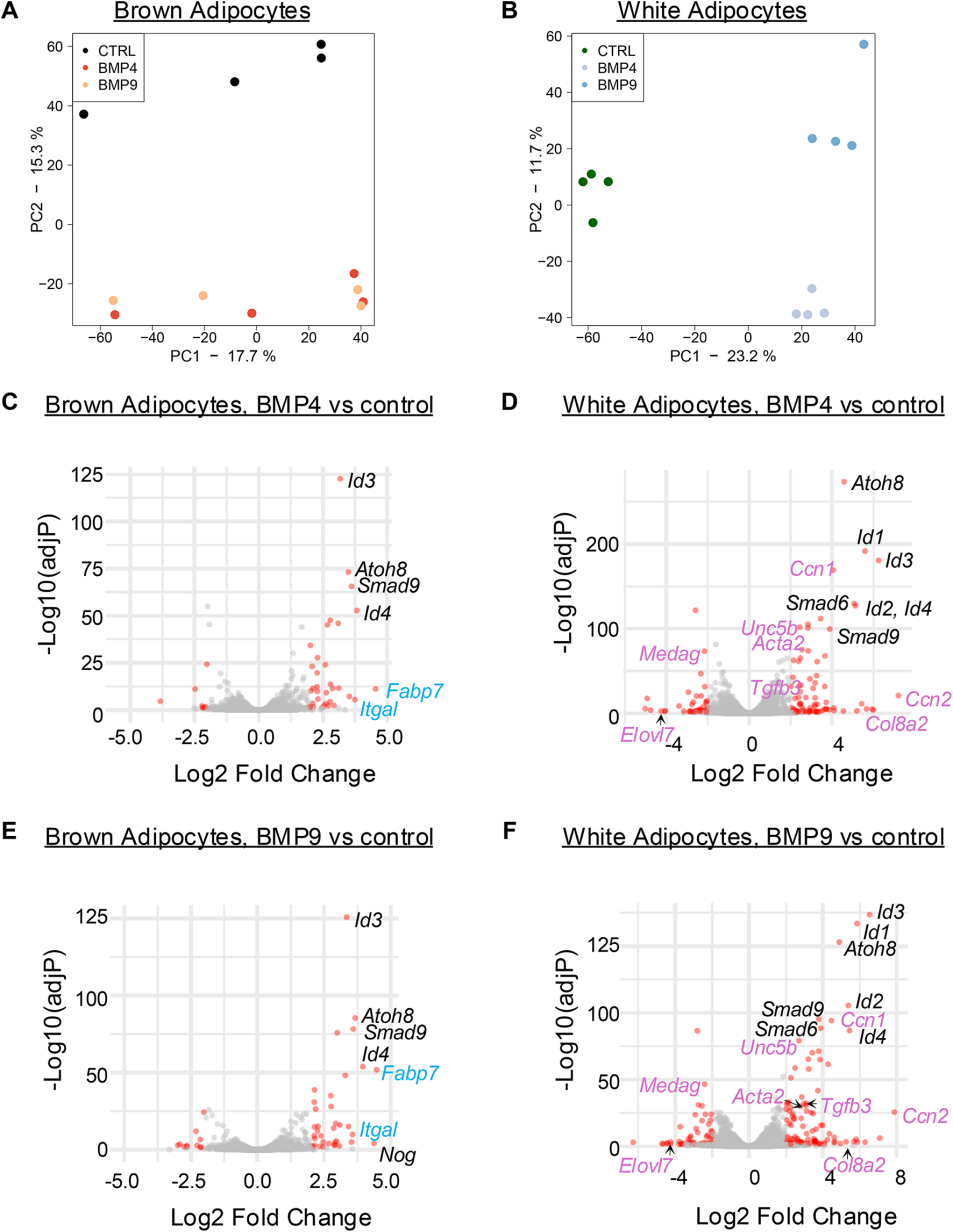
Overview of the RNAseq experiment comparing BMP4 and BMP9 signalling in brown and white adipocytes. (**A** and **B**) Principal component analysis (PCA) showing the clustering effects of BMP treatment. (**C** and **D**) Volcano plots of the differentially expressed genes (DEGs) comparing BMP4 treatment versus vehicle control in brown (**C**) and white (**D**) adipocytes. (**E** and **F**) Volcano plots of the DEGs comparing BMP9 treatment versus vehicle control in brown (**E**) and white (**F**) adipocytes. In (**C**–**F**) Red dots represent genes with adjusted *P*-values < 0.05 and Log2 Fold Changes greater than 2 or less than − 2. Canonical BMP target genes are labelled in black, whereas those only in brown labelled in cyan, and those only in white labelled in pink.

To evaluate the overall signalling outcome of BMP4 and BMP9 in mature brown and white adipocytes, RNAseq data was measured from adipocyte samples treated with BMP4, BMP9 or vehicle controls for 8 h. Post batch-effect correction, the principal component analysis (PCA) showed clear separation of BMP treatments versus controls in both adipocytes (Fig. 3A and B), with clear separation of BMP4- and BMP9-treatments only in white adipocytes (Fig. 3B), confirming that both BMPs exert significant effects on both brown and white adipocytes.

Volcano plots of differentially expressed genes (DEGs) showed that both BMP4 and BMP9 induced canonical BMP target genes in both adipocytes as observed in many other cell types^30,31^, such as *Id1-4*, *Atoh8*, *Smad9* (Fig. 3C–F), but they induced many more DEGs in mature white adipocytes (Fig. 3D,F) than in brown adipocytes (Fig. 3C,E). Among the DEGs present only in the white adipocytes, *Medag* is predicted to be involved in fat cell differentiation and *Elovl7* is involved in long-chain fatty acid elongation and remodelling, both of which were suppressed by BMP4 and BMP9 treatments. Noticeably, *Tgfb3,* a stimulator of adipocyte progenitor proliferation^32^, was upregulated by both BMPs in white adipocytes and several TGF-β target genes were observed, such as *Acta2* and *Ccn2* (also called *Ctgf*).

### Correlation analysis of BMP4 and BMP9 signalling in the same adipocytes, and the same BMP signalling in brown and white adipocytes

BMP4 and BMP9 signal via different type I receptors. Having shown that they signal with similar potency in mature adipocytes, we next asked whether BMP4 and BMP9 regulate a similar set of genes in the same adipocytes and whether each BMP regulates a similar or different set of genes in brown and white adipocytes.

**Fig. 4:**
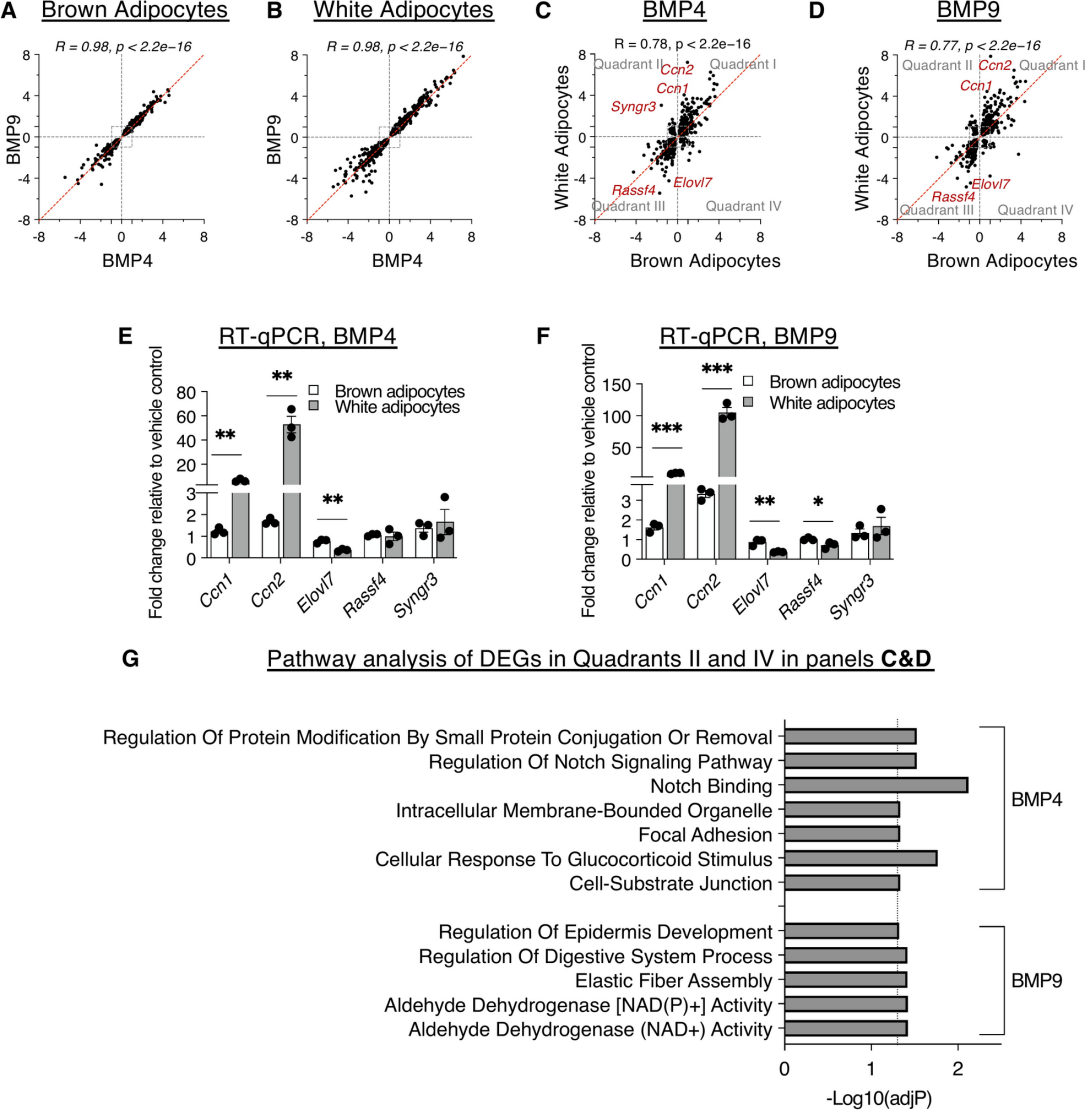
Correlation analyses comparing BMP4 and BMP9 signalling in the same adipocytes and the same BMP treatment in brown and white adipocytes. (**A** and **B**) Log2 Fold Changes of DEGs from BMP9 treatment plotted against those from BMP4 treatment in brown (**A**) and white (**B**) adipocytes, respectively. (**C**) Log2 Fold Changes of DEGs from BMP4-treated white adipocytes plotted against those from BMP4-treated brown adipocytes. (**D**) Log2 Fold Changes of DEGs from BMP9-treated white adipocytes are plotted against those from BMP9-treated brown adipocytes. (**E** and **F**) RT-qPCR validation of selected target genes highlighted in (**C** and **D**) from three independently differentiated brown and white adipocytes treated with BMP4 (**E**) or BMP9 (**F**). (**G**) Pathway analysis (using EnrichR) of genes in Quadrant II and IV in (**C** and **D**) which are the genes regulated in the opposite directions in brown and white adipocytes. In (**A**–**D**) red dashed lines are at a 45-degree angle, highlighting that DEGs on this line will have equal fold changes in both treatment conditions. Dotted grey boxes have the values of X =  ± 1 or Y =  ± 1. In (**E** and **F**) means ± SEM are shown, data are from three independently differentiated brown or white adipocytes. Two-tailed T-test for each gene, *, *P* < 0.05, **, *P* < 0.01, ***, *P* < 0.001.

Firstly, we performed correlation analyses of the DEGs induced by BMP4 versus BMP9 in brown and white adipocytes, respectively. As shown in Fig. 4A and B, BMP4 and BMP9 induced almost identical fold-changes of DEGs in mature brown or white adipocytes, respectively, with a correlation coefficient of 0.98 in both analyses. In contrast, the DEGs induced by each BMP in brown and white adipocytes have fewer but still significant overlaps, with correlation coefficients of 0.78 and 0.77 for BMP4 and BMP9, respectively (Fig. 4C,D). In quadrants I and III of Fig. 4C and D, substantially more DEGs deviate from the 45-degree line, appearing closer to the Y-axis. This observation suggests that for both BMP4 and BMP9 treatments, the shared genes have an overall bigger fold-changes difference (BMP vs Control) in the white adipocytes than in brown adipocytes. RT-qPCR analysis of independently differentiated brown and white adipocytes confirmed that for both BMP4 and BMP9 treatment, *Ccn1* and *Ccn2* mRNA induction is much higher in white than in brown adipocytes. Conversely, *Elovl7* is preferentially repressed only in white adipocytes (Fig. 4E,F). Interestingly, for both BMPs, there are DEGs present in Quadrants II and IV (Fig. 4C,D), which are regulated in opposite directions in brown and white adipocytes. Gene list enrichment analyses of the DEGs in Quadrants II and IV revealed the pathways and Gene Ontology (GO) terms that are differentially regulated in brown and white adipocytes (Fig. 4G), which include Notch signalling by BMP4, and aldehyde dehydrogenase activity by BMP9, respectively.

### Enriched pathways by BMP4 and BMP9 treatments in brown and white adipocytes

**Fig. 5:**
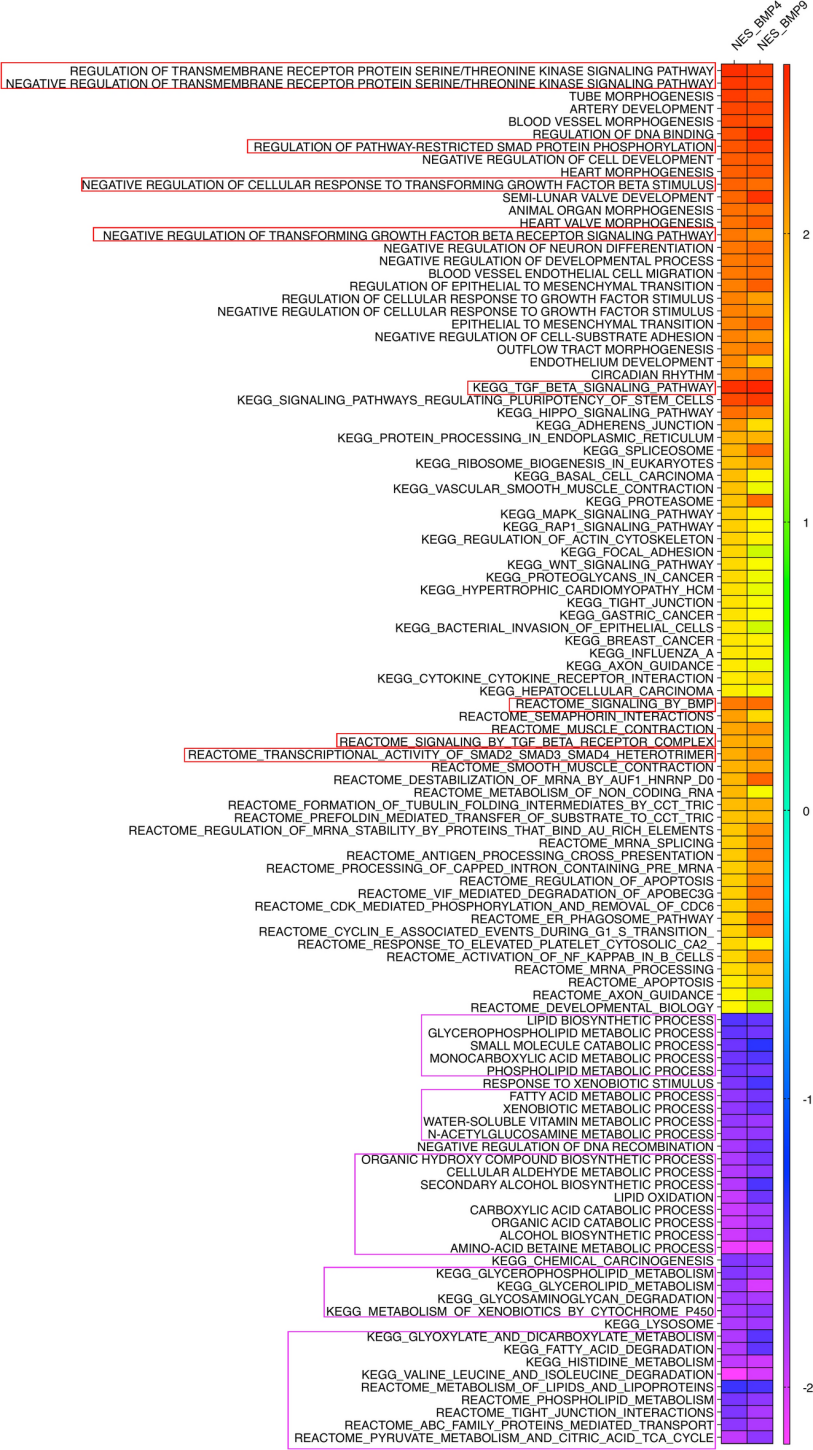
Gene Set Enrichment Analysis (using FGSEA) of DEGs in white adipocytes. Heatmap of normalised enrichment scores (NES) showing the top 25 upregulated GO terms, top 25 upregulated KEGG terms, top 25 upregulated Reactome terms, as well as all the downregulated terms. Red boxes highlight terms related to TGF-β signalling. Magenta boxes highlight terms related to lipid function and secondary metabolism.

Gene set enrichment analysis (GSEA) using the GO, KEGG^33,34^ and Reactome databases of the DEGs showed that in mature white adipocytes, for both BMP4 and BMP9, the number of upregulated terms was substantially higher than the down-regulated terms (Supplementary Fig. 2). Notably, there are significant overlaps between the BMP4 and BMP9 enrichment results. Figure 5 shows the normalised enrichment scores (NES) of the top 25 upregulated GO, KEGG and Reactome terms and all down-regulated terms according to the enrichment analysis. Many upregulated terms are related to TGF-β signalling and other signalling pathways, as well as different organ morphogenesis (Fig. 5). Interestingly, among the down-regulated GO, KEGG and Reactome terms, the majority are related to secondary metabolism and lipid metabolism, suggesting that both BMP4 and BMP9 signalling in mature white adipocytes suppresses white adipocytes metabolism and their functions. Our GSEA analysis did not reveal any significantly changed terms induced by BMP4 and BMP9 in mature brown adipocytes.

**Fig. 6:**
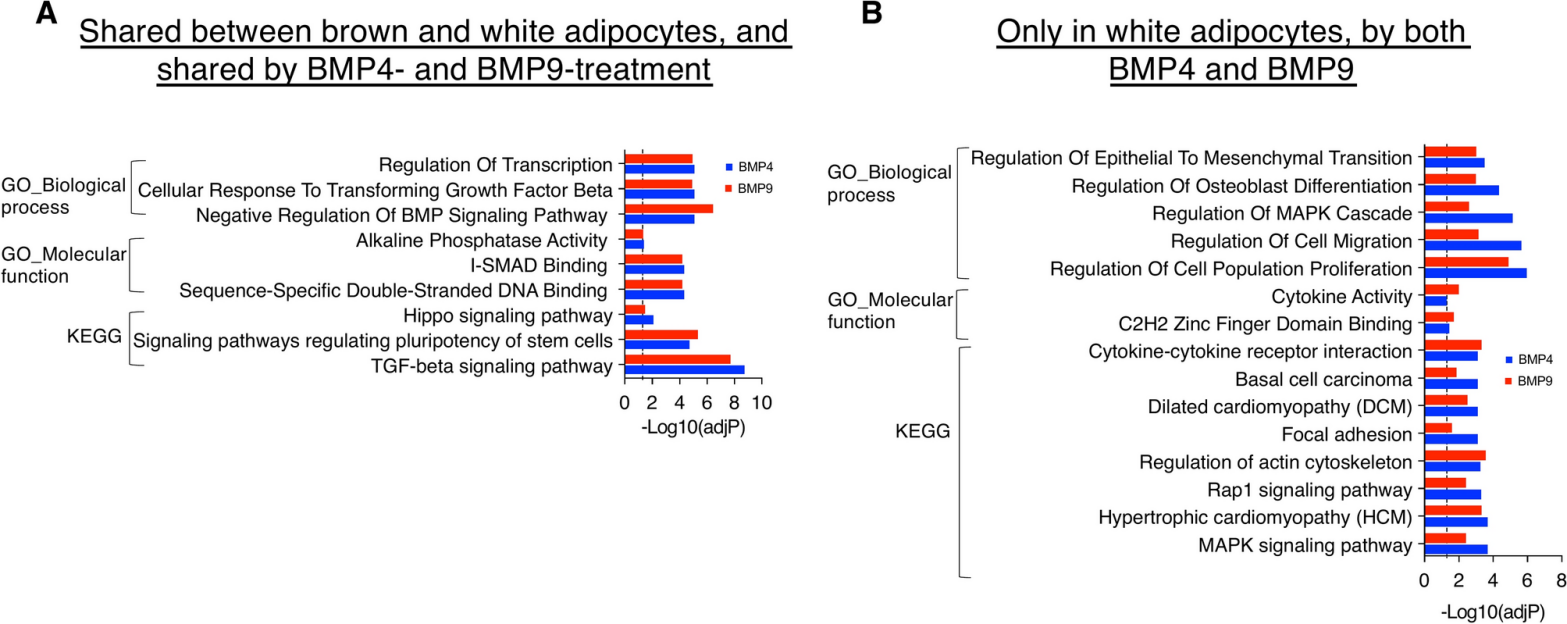
Enriched pathway analysis of DEGs using EnrichR from BMP4- and BMP9-treated brown and white adipocytes. (**A**) A representative selection of the most significantly enriched pathways shared between both brown and white adipocytes and shared by BMP4 and BMP9 treatment (from Rows 1 and 2 in Supplementary Fig. 3A). (**B**) A representative selection of significantly enriched pathways only in white adipocytes and shared by both BMP4 and BMP9 treatments (from Rows 3 and 5 in Supplementary Fig. 3A). Grey dotted lines have X = 1.3 which represent the cutoff point for an adjusted *P*-value of 0.05.

Next, we performed gene list enrichment analysis using EnrichR to investigate the common and differentially enriched pathways following BMP4 or BMP9 treatments in brown and white adipocytes, respectively. EnrichR is an alternative tool for performing enrichment analysis on curated gene sets using a different computational approach to GSEA^35^. Supplementary Fig. 3A summarises the number of significantly enriched GO and KEGG terms by each BMP. Firstly, many significantly enriched pathways are shared between brown and white adipocytes and between BMP4 and BMP9 treatments (Fig. 6A). Such pathways include regulation of transcription, cellular response to TGF-β and BMP signalling, alkaline phosphatase activity, and signalling pathways regulating pluripotency of stem cells. Many significantly enriched pathways are present only in white adipocytes and shared by both BMPs (Fig. 6B), which include regulation of epithelial to mesenchymal transition, regulation of osteogenic differentiation, regulation of MAPK cascade, regulation of cellular migration and proliferation. Interestingly, in white adipocytes, there are a few pathways that are only enriched by BMP4 (Supplementary Fig. 3A and B) but not by BMP9 treatment, including several terms related to the cell-substrate junction and regulation of cell–matrix adhesion, suggesting that BMP9 might induce a subset of BMP4 signalling in white adipocytes. In contrast, BMP9 signalling in brown adipocytes can enrich pathways that are not seen for BMP4 (Supplementary Fig. 3A and C), including regulation of FGF signalling and Calcium signalling, suggesting BMP9 may have a non-overlapping role to BMP4 in brown adipocytes.

### Transcription factors regulated by BMP signalling in brown and white adipocytes

**Fig. 7:**
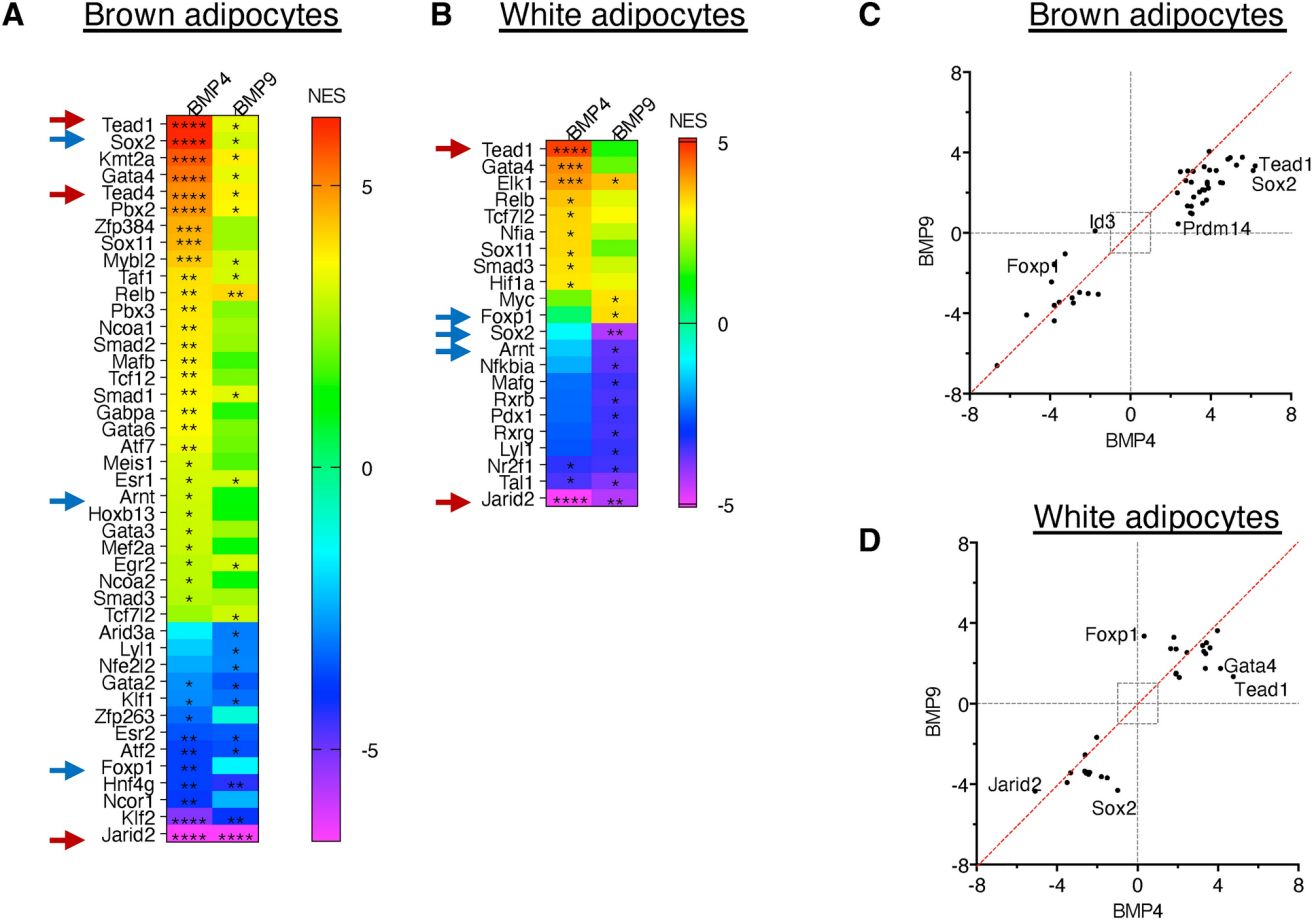
Transcription factor activity analysis of DEGs using Virtual Inference of Protein-activity by Enriched Regulon (VIPER). (**A** and **B**) Heatmaps of significantly changed transcription factor activities from BMP4- or BMP9-treated brown (**A**) and white (**B**) adipocytes. (**C** and **D**) Correlation analysis of the transcription factors identified using normalised enrichment score (NES) from BMP4- or BMP9-treated brown (**C**) and white (**D**) adipocytes. In (**A** and **B**) *, FDR < 0.05, **, FDR < 0.01, ***, FDR < 0.001, **** FDR < 0.0001. In (**C** and **D**) red dashed lines are at a 45-degree angle, highlighting that points on this line have equal NES values in both BMP4- and BMP9-treatment. Dotted grey boxes have X =  ± 1 or Y =  ± 1.

As DNA binding proteins and transcription factors are highly regulated by BMP signalling, we performed transcription factor activity analysis using VIPER to investigate the transcriptional activities initiated by BMP4 and BMP9, respectively. Interestingly, we detected significantly more changes in transcription factor activities in brown adipocytes than in white adipocytes. In the brown adipocytes, more positive normalised enrichment scores (NES) were significantly induced by BMP4 than by BMP9, yet a similar number of negative NES were induced by both BMP4 and BMP9 (Fig. 7A). In white adipocytes, we observed fewer overlaps between BMP4 and BMP9, with more significant positive NES induced by BMP4 and more significant negative NES induced by BMP9 (Fig. 7B).

Some changes were consistent between brown and white adipocytes. For example, Jarid2 has a significant negative NES in both BMP4 and BMP9 treatment and in both adipocytes. Jarid2 is a cofactor for polycomb repressive complexes (PRC) and plays a role in the crosstalk between histone modification and PRC2 activity^36^. Down-regulation of Jarid2 activity suggests an upregulation of gene transcriptional activity in both adipocytes following both BMP treatments. In addition, both Tead1 and Gata4 transcriptional activities were among the most significantly upregulated hits by BMP4 in both adipocytes.

In brown adipocytes, many NES changes are shared by BMP4 and BMP9. For example, Tead1 and Tead4, which are the final nuclear effectors of the Hippo pathway, are positively enriched by both BMPs. Similar significant positive NES shared by BMP4 and BMP9 include Sox2, Kmt2a, Gata4, Pbx2, Mybl2, Taf1, Relb, Smad1, Esr1 and Egr2. Apart from Jarid2, significant negatively enriched transcription factors in both BMPs include Klf2, Hnf4g, Arf2, Esr2, Klf1 and Gata2. In white adipocytes, BMP4 regulated more transcriptional activities with positive NES, such as Tead1, Gata4, Relb, Sox11, Smad3 and Hif1a. In contrast, BMP9 regulated more transcriptional activities with negative NES, which included Arnt, Nfkbia,Mafg, Rxrb and Pdx1.

Three transcription factors are differentially regulated by BMP4 and BMP9 in brown and white adipocytes. Sox2 activity, which is upregulated by both BMPs in brown adipocytes, is significantly downregulated by BMP9 in white adipocytes. Arnt is significantly upregulated by only BMP4 in brown adipocytes but significantly downregulated by only BMP9 in white adipocytes. The third one is Foxp1, which is significantly downregulated by BMP4 in brown adipocytes yet upregulated by only BMP9 in white adipocytes.

Correlation analysis of the transcription factor activity revealed a similar picture (Fig. 7C,D) in that BMP4 and BMP9 regulate many overlapping transcription factors in similar directions. Overall, there are more extensive changes induced by BMP4 in brown adipocytes, such as Tead1 and Sox2 with bigger positive NES and Foxp1 and Id3 with more negative NES. In contrast, in white adipocytes, Foxp1 and Sox2 lie much closer to the Y-axis and in opposite directions, which means that their activities are more enriched after BMP9 treatment (Fig. 7D).

### BMP4 and BMP9 induced gene expression changes in multiple extracellular regulators of the TGF-β superfamily in mature brown and white adipocytes

Intriguingly, one of the most significantly enriched pathways induced by both BMPs in both adipocytes is the TGF-β signalling (Fig. 6A). It is well-known that the extracellular regulation of TGF-β and BMP signalling is interconnected and has a high degree of promiscuity and complexity^37,38^. To gain further insight into how BMP4 and BMP9 treatments could change the dynamics of the extracellular regulation of the TGF-β signalling, we performed further detailed analyses of genes encoding the ligands, receptors, co-receptors and ligand traps.

**Fig. 8:**
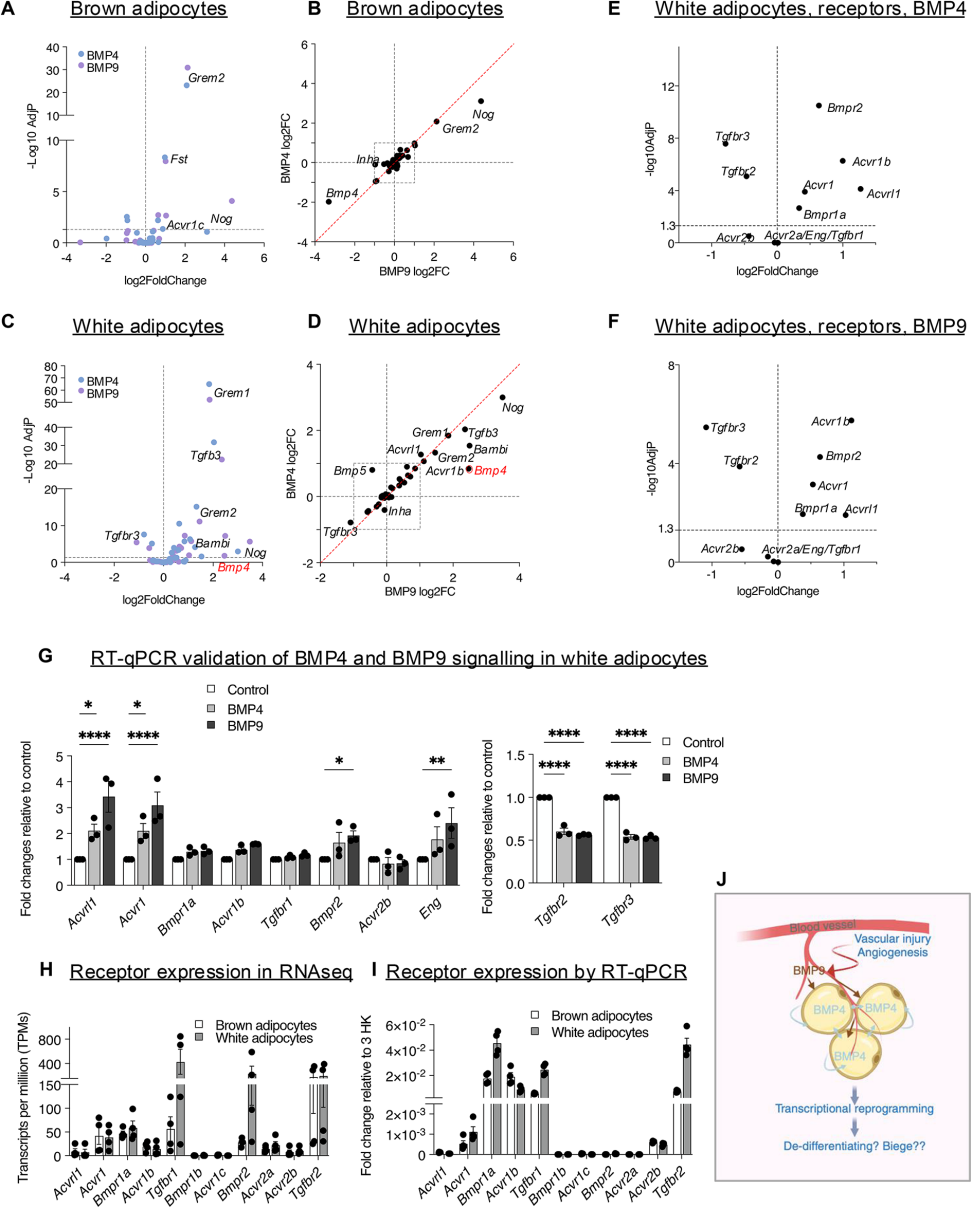
BMP4 and BMP9 suppress BMP and activin signalling in brown adipocytes but initiate reprogramming of TGF-β family signalling in white adipocytes. Genes encoding TGF-β family ligands, receptors, co-receptors and ligand traps were selected from the DEG list and further analysed in volcano plots (**A** and **C**) and correlation plots (**B** and **D**). Note that only those that are detected in the RNAseq datasets are included. (**A** and **C**) Volcano plots of BMP4- and BMP9-induced DEGs in brown (**A**) and white adipocytes (**C**). (**B** and **D**) correlation plots analysing the DEG Log2 Fold Changes induced by BMP4 and BMP9 in brown (**B**) and white (**D**) adipocytes. (**E** and **F**) BMP4 (**E**) and BMP9 (**F**) induce significant changes in the TGF-β family receptors in white adipocytes, upregulating *Bmpr2* as well as four type I receptors which mediate signals from BMPs, activins and growth and differentiation factors (GDFs), but suppressing type II and co-receptors for TGF-β signalling. (**G**) RT-qPCR validation of receptors changes in E&F, using three independently differentiated brown and white adipocytes treated with control or BMPs. Means ± SEM are shown. Two-way ANOVA. *, *P* < 0.05, **, *P* < 0.01, ****, *P* < 0.0001. (**H**) Receptor expression levels in brown and white adipocytes from the transcripts per million (TPMs) of the RNAseq datasets, vehicle-treated samples. (**I**) The same receptors were analysed by RT-qPCR from independently differentiated brown and white adipocytes, serum-starved medium overnight and treated with vehicle for 8 h. Means ± SEM are shown. (**J**) Model summarising BMP4 and BMP9 signalling in white adipocytes. Circulating BMP9 can act on white adipocytes, probably upon angiogenesis process or vascular injury, and can induce a similar set of genes to BMP4 as well as the BMP4 gene itself. BMP4 is produced by the white adipocytes and can act in an autocrine or paracrine manner. The overall effect of BMP4 and BMP9 signalling in white adipocytes activates a new program of TGF-β family signalling and transcriptional response, suppressing the metabolic function of adipocytes, likely to transform the mature adipocytes to a more progenitor-like form. Created in https://BioRender.com.

In brown adipocytes, 47 genes could be detected in the dataset. Both BMP4 and BMP9 showed remarkably similar DEGs in genes of the TGF-β signalling machinery, with identical genes being most significantly regulated (Fig. 8A) and well-correlated fold changes among all genes upon treatments (Fig. 8B). In both BMP4- and BMP9-treated brown adipocytes, the most significantly upregulated genes were *Grem2* and *Fst* (Fig. 8A), and the biggest fold changes of the upregulated genes were *Nog* and *Grem2* (Fig. 8B). These genes encode Gremlin2, Follistatin and Noggin, respectively; all are extracellular inhibitors of signalling from BMPs, activins or growth and differentiation factors (GDFs). Such results strongly suggest that both BMP4 and BMP9 have similar effects in suppressing the TGF-β family signalling in brown adipocytes.

In white adipocytes, 45 genes could be measured in the dataset, out of which, 15 genes were significantly changed upon BMP9 treatment and 14 upon BMP4 treatment (Fig. 8C–F). The most significantly upregulated genes were *Grem1*, *Tgfb3* and *Grem2*, and the most significantly downregulated gene was *Tgfbr3*. Again, fold changes of the DEGs regulated by BMP4 and BMP9 showed good correlation apart from one exception, namely, *Bmp4*, which showed bigger fold induction by BMP9. Although the downregulation of *Bmp5* was only detected in BMP9 treatment (Fig. 8D), such a change was not statistically significant.

The expression levels of several receptors and co-receptors were significantly changed in white adipocytes after BMP4 and BMP9 treatments (Fig. 8E and F), with 4 type I receptors mediating the BMP and activin/GDF signalling being upregulated. RT-qPCR analysis of independently differentiated white adipocytes treated with BMP4 or BMP9 confirmed the changes in *Acvrl1*, *Acvr1*, *Bmpr2*, *Eng*, *Tgfbr2* and *Tgfbr3* (Fig. 8G). This was in contrast with brown adipocytes where only *Acvr1c* was significantly induced by both BMP4 and BMP9 treatment (Supplementary Fig. 4). This suggests that in white adipocytes, both BMP4- and BMP9-treatments initiate a similar and significant shift and reprogramming of the TGF-β family signalling. To understand the basal expression levels of the receptors and to evaluate which receptors could mediate the BMP4 and BMP9 signalling, we examined the mRNA levels of the receptors and co-receptors in the brown and white adipocytes under basal serum-starved condition before applying BMP treatments, using either RNAseq data or independent RT-qPCR validation samples. From the RNAseq vehicle-treated samples, we saw high expression of *Bmpr1a, Tgfbr1* and *Tgfbr2* in both brown and white adipocytes, followed by *Acvr1, Acvr1b* and *Bmpr2* (Fig. 8H). Such expression pattern was mostly replicated by the qPCR measurements of these receptors (Fig. 8I). There are discrepancies of *Acvr2a*, *Acvr2b* and* Bmpr2* expression levels between the RNAseq and qPCR data, which could be due to multiple reasons including the different serum-starvation time as both *Bmpr2* and *Acvr2b* are regulated by BMP9 which is present in serum. Nevertheless, the high expression levels of type I receptor *Bmpr1a* followed by *Acvr1* support that both adipocytes will be highly responsive to BMP4 and BMP9 treatment. BMP4 is known to use BMPRIa as the type I receptor. BMP9 is most likely to signal via ACVR1 initially, and via both ACVRL1 and ACVR1 after induction of *Acvrl1* by BMPs or in complete growth medium which contains BMP9. Both adipocytes are expected to be highly responsive to TGF-β treatment, as both *Tgfbr1* and *Tgfbr2* are highly expressed.

## Discussion

In this study, we compared BMP4 and BMP9 signalling in mature brown and white adipocytes in dose–response and time-course assays as well as by global gene expression using RNAseq analysis. We found that overall, white adipocytes were more responsive to BMP4 and BMP9 treatments than brown adipocytes, resulting in a higher number of regulated genes and pathways. Two most unexpected results were: (1) the downregulation of many metabolic pathways by both BMPs (Fig. 5); (2) the significant changes in the dynamics of the TGF-β family signalling (Fig. 8) being preferentially observed in white adipocytes.

Although BMP4 is a secreted growth factor, when expressed in mammalian cells, it was mainly associated with cell surface extracellular matix^39^. In fact, it was also observed in Xenopus embryos that the BMP4 growth factor domain was trapped in the ECM^40^. The observation that BMP9 induces *Bmp4* expression in white adipocytes suggests one possibility that some effects of BMP9 treatment, especially after prolonged treatment, could be coming from BMP4 (Fig. 8J). The observations that BMP9 regulates a subset of BMP4 response in the functional enrichment analysis (Supplementary Fig. 3A) and that *Bmp4* is the only significantly differentially regulated ligand between BMP4- and BMP9-treatment (Fig. 8C and D) lend support to this possibility.

Less regulated pathways observed in brown adipocytes are consistent with the observation that most upregulated TGF-β related genes are ligand traps and inhibitors (Fig. 8A and B), which means that by 8 h, most primary effects from BMP signalling may have already disappeared. Future RNAseq experiments with shorter treatment times may reveal direct target genes by different BMPs. Nevertheless, the current data suggest that BMP4 and BMP9 may have less impact on brown adipocyte function than on white adipocytes after prolonged exposure.

BMP4 is highly expressed and enriched in white adipose tissues in humans and mice^14^. Serum levels of BMP4 in human subjects correlate positively with adipocyte diameter and negatively with insulin sensitivity^14^. Overexpression of the BMP4 gene in white adipocytes in mice led to reduced WAT mass and white adipocyte size^18^. Our observation that BMP4 and BMP9 suppress white adipocyte metabolism is consistent with these reports and suggests that BMP9 may also participate in white adipocyte plasticity and remodelling.

BMP9 is primarily synthesised in the liver and accounts for most BMP activities in circulation^41^. Adipose tissues are highly vascularised and BMP9 may directly encounter adipocytes at adipose tissue depots upon vascular injury or angiogenesis. Our data that BMP9 signals actively in white adipocytes, induces a similar response to BMP4 and upregulates the expression of BMP4 itself is consistent with the in vivo study that BMP9 administration reduced the size of white adipocytes like BMP4^16^. Future work using a knockout mouse coupled with a vascular injury model could help to address whether BMP9 plays a role in modulating BMP4 signalling in WAT.

Our data supports a working model of BMP4 and BMP9 signalling in white adipocytes as shown in Fig. 8J. Circulating BMP9 can act on white adipocytes, probably upon angiogenesis process or vascular injury, and can induce BMP4 expression. BMP4 is produced by the white adipocytes and can act in an autocrine or paracrine manner. The overall effect of BMP4 and BMP9 signalling in white adipocytes activates a new program of TGF-β family signalling and transcriptional response, suppressing the metabolic function of adipocytes, likely to transform the mature adipocytes to a more progenitor-like form.

This snapshot and comprehensive analysis of BMP4 and BMP9 signalling in mature brown and white adipocytes also have essential impacts from the understanding of BMP and TGF-β signalling mechanisms standpoint. Here we provided detailed data and direct evidence to show that: (1) many aspects of the BMP signalling are conserved across different cells and different BMPs, such as the induction of the canonical BMP target genes in both adipocytes and the shared enriched pathways by both BMPs and in both adipocytes; (2) although BMP4 and BMP9 utilise different type I receptors, they regulate exceptionally similar set of genes in the same cells, with almost identical fold changes. This demonstrates that as far as the cellular contexts (i.e., intracellular mediators) and signalling strength are equivalent, the signalling outcome (i.e., the downstream DEGs) will be similar and could be independent of the type I receptors; (3) context-dependent signalling is exemplified in that the identical BMP can regulate different genes in different adipocytes, despite brown and white adipocytes sharing many similarities; (4) this study reinforces the concept that BMP signalling induces auto-regulation loops. In addition to the regulation of different Smad proteins, particularly *Smad6* and *Smad7* being part of the most highly induced canonical BMP target genes, in brown adipocytes, BMP4 and BMP9 induced Gremlins, Noggin and Follistatin, thereby turning down BMP signalling. In contrast, in white adipocytes, BMP signalling was amplified and reprogrammed by the induction of different type I receptors and ligands.

Signal transduction machineries mediating different TGF-β family ligands are interconnected. In fact, it is well-documented that many other TGF-β family ligands are also involved in adipogenesis and adipocyte’s function^42,43^. A recent report used a 3T3-L1 differentiation assay to screen different TGF-β family ligands, ligand traps and small molecule inhibitors in adipogenesis and identified several ligands in the activin and TGF-β branch that could suppress 3T3-L1 adipogenesis^44^. BMP2 and BMP6 signalling in white adipocytes led to enhanced insulin sensitivity in a PPARγ-dependent manner^45^. The importance and interconnection of BMP signalling in adipose tissue are highlighted by the report that genetic ablation of *Bmpr1a* in brown adipogenic progenitor cells led to the loss of brown adipose tissue and compensatory browning of the white fat^46^.

This study also has implications for targeting the BMP signalling for therapeutic applications, for example, targeting BMP9 signalling^47^ and other TGF-β family ligands^48,49^ is a hot area for novel drug discovery and development for pulmonary arterial hypertension^50^. Subcutaneous injection is a popular route for the administration of biologic drugs. The data in this study would be very informative when designing novel biologics targeting the BMP and TGF-β signalling complexes. For example, it would be important to consider whether the BMP-modifying biologics affect the local adipocyte’s function in a desired or undesired way and whether the adipocytes could consume such biologics.

In summary, we have provided direct evidence that BMP9 signals potently in mature brown and white adipocytes with similar outcome to BMP4. Importantly, both BMPs suppressed essential metabolic pathways and initiated reprogramming of TGF-β family signalling only in white adipocytes which may contribute to the plasticity of the white adipocytes.

## Methods

### P-BAT murine brown pre-adipocyte cell line

The murine P-BAT brown pre-adipocyte immortalised cell line, which can be differentiated into mature brown adipocytes in vitro^9,51^, was generously provided by Prof. Angela Valverde from Instituto de Investigaciones Biomédicas Sols-Morreale (IIBM), Madrid, Spain. This cell line was generated initially from the stromal vascular fraction (SVF) cells of interscapular BAT from newborn mice, with the sexes of the mice not reported.

P-BAT cells were cultured in DMEM (Catalogue No. 11965092, Gibco), supplemented with 10% heat-inactivated FBS (Catalogue No. A5256701, Gibco) and 1% Pen-Strep solution (Catalogue No. P0781, Sigma). The cultures were maintained at 37 °C in a humidified incubator with a 5% CO₂. For differentiation, cells were seeded in 6-well plates at a density of 1.5 × 10^5^ cells per well. The growth medium was supplemented with 1 nM T3 and 20 nM insulin until the cells reached 75%–80% confluence. At this stage, differentiation was induced by adding IBMX (500 μM), dexamethasone (2 μM), and indomethacin (125 μM) to the medium for 24 h. Subsequently, the medium was replaced with the original growth medium containing only T3 and insulin, which was refreshed every 48 h. Complete differentiation was typically achieved in approximately 8–9 days.

### 3T3-L1 murine white pre-adipocyte cell line

The 3T3-L1 cell line was acquired from the American Type Culture Collection (ATCC CL-173). 3T3-L1 cells were cultured in DMEM, supplemented with 10% heat-inactivated FBS and 1% Pen-Strep solution. The cultures were maintained at 37 °C in a humidified incubator with a 5% CO₂. For differentiation, the cells were seeded in 6-well plates in DMEM supplemented with 10% FBS and 1% Pen-Strep solution at a density of 2.5 × 10^5^ cells per well. Once 100% confluence was achieved, differentiation was initiated by adding IBMX (0.5 mM), dexamethasone (1 μM), and insulin (5 μg/ml) to the medium. 72 h post-induction, IBMX and dexamethasone were removed from the medium, while insulin supplementation continued for an additional six days. This medium was changed every two days. Complete differentiation typically occurs within 12 days.

### Signalling assays

After brown and white adipocytes were differentiated and around 16 h before signalling assays, cells were washed with PBS and switched to quiescence media which comprised DMEM supplemented with 0.1% FBS and 1% Pen-Strep. The concentrations of each BMP treatment and the duration of the treatment are detailed in figure legends. Following the treatment period, the cells were harvested for either western blots or quantitative PCR analysis. Western blot analysis of the cell lysates involved non-reducing SDS-PAGE using 40 μg of total protein, and subsequent immunoblotting with anti-phosphoSMAD1/5 antibodies.

### RNA extraction from cells and quantitative real-time polymerase chain reaction (qRT-PCR)

The RNA extraction was performed using the RNeasy Plus Mini Kit (Qiagen, 74104) according to the instructions provided by the manufacturer. A high-capacity reverse-transcriptase kit (Applied Biosystems) facilitated the synthesis of cDNA from 1 μg of RNA per sample, using random primers, in line with the manufacturer’s instructions. qRT-PCR analyses were conducted in 384-well plates (Applied Biosystems). Each well contained a 10 μl reaction mixture, comprising 2 μl of tenfold diluted cDNA and 8 μl of PowerUP™ SYBR Green PCR master mix (Applied Biosystems, A25742), which included both forward and reverse primers at a final concentration of 200 nM each. The primer sequences utilised are detailed in Supplementary Table 1. The PCR cycling started with a 2-min denaturation step at 95 °C, followed by 50 cycles, each consisting of 95 °C for 30 s, 55 °C for 30 s, and 72 °C for 30 s. These reactions were executed using a QuantStudio 6 system (Applied Biosystems), which automatically calculated the cycle threshold (Ct) values for each well. All samples were tested in triplicate to ensure reproducibility; the results were then averaged for analysis. Gene expression levels were normalised to the geometric mean of three housekeeping (HK) genes: β2-microglobulin (B2M), β-actin, and HPRT. The data are presented as fold-change relative to control samples, calculated using the 2^-ΔΔCt method unless specified otherwise.

### RNA sequencing (RNAseq)

Four independent batches of brown pre-adipocytes and four independent batches of white pre-adipocytes were differentiated. They were subsequently treated with 3 ng/ml of BMP4 or BMP9 for 8 h, along with vehicle controls. Total RNA from these cells was extracted as above using the RNeasy Plus Mini Kit (Qiagen, 74,104). The integrity and quality of the extracted RNA were assessed using an Agilent Bioanalyzer. Library preparation and RNAseq were performed at Novogene. The sequencing process was carried out using an Illumina NovaSeq 6000 system.

FASTQC (v0.11.9) was used to generate quality-control reports of individual FASTQ files (http://www.bioinformatics.babraham.ac.uk/projects/fastqc). The reads were aligned to the mus musculus GRCm38 reference genome using Hisat2 (v2.1.0)^52^ with default parameters, and the genes were counted with HTSeq2 (v0.11.1)^53,54^. BiomaRt package^54^ was used to map ensembl_gene_ids to gene_symbols. Batch effect correction was performed using Bioconductor’s function “COMBAT”^55^ (from package “sva”) on the quantile normalised counts. Differential gene expression analysis was performed with DESeq2 (v1.26.0)^56^ and the Benjamini–Hochberg method was applied to adjust the raw p-values to control the False Discovery Rate (FDR). Pathway enrichment was assessed using both FGSEA (Fast gene set enrichment analysis)^57^ and EnrichR^35^. Transcriptional regulator activity was assessed using VIPER (Virtual Inference of Protein-activity by Enriched Regulon analysis)^58^.

### Oil red O lipid staining

Adipocyte lipid droplets were visualised using Oil Red O staining. A 0.25% stock solution was made by dissolving 0.25 g Oil Red O powder (Sigma, O0625) in 100 ml isopropanol and incubating at 56 °C for 1 h. A fresh working solution of Oil Red O was created by mixing six parts of the stock solution with four parts deionised water. This mixture was vigorously shaken and allowed to stand for 10 min, then filtered through a 0.45 μm syringe filter. Prior to staining, the adipocytes were fixed in 10% formalin (Sigma, HT501128) for 1 h at room temperature, followed by two additional PBS washes. Post-fixation, the cells were treated with 60% isopropanol for 5 min to facilitate the uptake of the dye. The prepared Oil Red O working solution was then applied to the cells and left to stain at room temperature for 30 min. After staining, the cells underwent one wash with 60% isopropanol and two further washes with PBS to remove excess dye and enhance the clarity of the lipid droplets. The stained adipocytes were finally visualised and imaged using an EVOS M5000 microscope (Invitrogen).

### Statistics

All experimental data are presented as the mean ± standard error of the mean (SEM). The specific number of experimental replicates is detailed in the accompanying figure legends. When data are presented as fold-changes, the baseline normalisation value is explicitly indicated in the figure legends. The selection of statistical tests was tailored to the experimental design. Comparisons between two groups were assessed using two tailed Student’s t-test. One-way analysis of variance (ANOVA) was applied for analyses involving more than two groups, incorporating Tukey’s post-hoc test for multiple comparisons. A threshold of *P* < 0.05 was predefined to establish statistical significance. All statistical analyses were conducted utilising GraphPad Prism (version 8.3.0 for Mac OS, GraphPad Software, San Diego, California, USA). The statistical approach employed and the demarcations of significance are explicitly noted in the figure legends.

## Supplementary Information

Supplementary Information.

## Data Availability

RNAseq raw data and processed gene expression files are publicly available through the Array Express portal (https://www.ebi.ac.uk/biostudies/arrayexpress/studies/E-MTAB-14609)

## References

[1] Longo, M. et al. Adipose tissue dysfunction as determinant of obesity-associated metabolic complications. *Int. J. Mol. Sci.* **20**, 2358 (2019). 31085992 10.3390/ijms20092358 PMC6539070

[2] Petito, G. et al. Adipose tissue remodeling in obesity: An overview of the actions of thyroid hormones and their derivatives. *Pharmaceuticals (Basel)* **16**, 572 (2023). 37111329 10.3390/ph16040572 PMC10146771

[3] Slawik, M. & Vidal-Puig, A. J. Adipose tissue expandability and the metabolic syndrome. *Genes Nutr.* **2**, 41–45 (2007). 18850138 10.1007/s12263-007-0014-9 PMC2474894

[4] Cinti, S. The adipose organ. *Prostaglandins Leukot. Essent. Fatty Acids* **73**, 9–15 (2005). 15936182 10.1016/j.plefa.2005.04.010

[5] Lenz, M., Arts, I. C. W., Peeters, R. L. M., de Kok, T. M. & Ertaylan, G. Adipose tissue in health and disease through the lens of its building blocks. *Sci. Rep.* **10**, 10433 (2020). 32591560 10.1038/s41598-020-67177-1 PMC7319996

[6] Nanduri, R. et al. Epigenetic regulation of white adipose tissue plasticity and energy metabolism by nucleosome binding HMGN proteins. *Nat. Commun.* **13**, 7303 (2022). 36435799 10.1038/s41467-022-34964-5 PMC9701217

[7] Kajimura, S. The epigenetic regulation of adipose tissue plasticity. *Proc. Natl. Acad. Sci. U. S. A.*10.1073/pnas.2102944118 (2021). 33741735 10.1073/pnas.2102944118 PMC8053925

[8] Gustafson, B. et al. BMP4 and BMP antagonists regulate human white and beige adipogenesis. *Diabetes* **64**, 1670–1681 (2015). 25605802 10.2337/db14-1127

[9] Miranda, S., Gonzalez-Rodriguez, A., Revuelta-Cervantes, J., Rondinone, C. M. & Valverde, A. M. Beneficial effects of PTP1B deficiency on brown adipocyte differentiation and protection against apoptosis induced by pro- and anti-inflammatory stimuli. *Cell. Signal.* **22**, 645–659 (2010). 20026400 10.1016/j.cellsig.2009.11.019

[10] Harms, M. J. et al. Mature human white adipocytes cultured under membranes maintain identity, function, and can transdifferentiate into brown-like adipocytes. *Cell. Rep.* **27**, 213–225 (2019). 30943403 10.1016/j.celrep.2019.03.026

[11] Cohen, P. & Kajimura, S. The cellular and functional complexity of thermogenic fat. *Nat. Rev. Mol. Cell Biol.* **22**, 393–409 (2021). 33758402 10.1038/s41580-021-00350-0 PMC8159882

[12] Reddi, A. H. BMPs: From bone morphogenetic proteins to body morphogenetic proteins. *Cytokine Growth Factor Rev.* **16**, 249–250 (2005). 15949967 10.1016/j.cytogfr.2005.04.003

[13] Blazquez-Medela, A. M., Jumabay, M. & Bostrom, K. I. Beyond the bone: Bone morphogenetic protein signaling in adipose tissue. *Obes. Rev.* **20**, 648–658 (2019). 30609449 10.1111/obr.12822 PMC6447448

[14] Modica, S. et al. Bmp4 promotes a brown to white-like adipocyte shift. *Cell Rep.* **16**, 2243–2258 (2016). 27524617 10.1016/j.celrep.2016.07.048

[15] Tseng, Y. H. et al. New role of bone morphogenetic protein 7 in brown adipogenesis and energy expenditure. *Nature* **454**, 1000–1004 (2008). 18719589 10.1038/nature07221 PMC2745972

[16] Kuo, M. M. et al. BMP-9 as a potent brown adipogenic inducer with anti-obesity capacity. *Biomaterials* **35**, 3172–3179 (2014). 24439409 10.1016/j.biomaterials.2013.12.063

[17] Um, J. H. et al. Bone morphogenic protein 9 is a novel thermogenic hepatokine secreted in response to cold exposure. *Metabolism* **129**, 155139 (2022). 35063533 10.1016/j.metabol.2022.155139

[18] Qian, S. W. et al. BMP4-mediated brown fat-like changes in white adipose tissue alter glucose and energy homeostasis. *Proc. Natl. Acad. Sci. U. S. A.* **110**, E798-807 (2013). 23388637 10.1073/pnas.1215236110 PMC3587258

[19] Huang, H. et al. Circulating bone morphogenetic protein-9 levels are associated with hypertension and insulin resistance in humans. *J. Am. Soc. Hypertens.* **12**, 372–380 (2018). 29550458 10.1016/j.jash.2018.02.007

[20] Luo, Y. et al. Decreased circulating BMP-9 levels in patients with type 2 diabetes is a signature of insulin resistance. *Clin. Sci. (Lond.)* **131**, 239–246 (2017). 27940998 10.1042/CS20160543

[21] Xu, X. et al. Circulating bone morphogenetic protein-9 in relation to metabolic syndrome and insulin resistance. *Sci. Rep.* **7**, 17529 (2017). 29235531 10.1038/s41598-017-17807-y PMC5727514

[22] Yang, Z. et al. CRISPR-mediated BMP9 ablation promotes liver steatosis via the down-regulation of PPARalpha expression. *Sci. Adv.*10.1126/sciadv.abc5022 (2020). 33246954 10.1126/sciadv.abc5022 PMC7695473

[23] Caperuto, L. C. et al. Modulation of bone morphogenetic protein-9 expression and processing by insulin, glucose, and glucocorticoids: Possible candidate for hepatic insulin-sensitizing substance. *Endocrinology* **149**, 6326–6335 (2008). 18703636 10.1210/en.2008-0655

[24] Kim, S., Choe, S. & Lee, D. K. BMP-9 enhances fibroblast growth factor 21 expression and suppresses obesity. *Biochim. Biophys. Acta* **1862**, 1237–1246 (2016). 27085971 10.1016/j.bbadis.2016.04.006 PMC5550097

[25] Yang, M. et al. Role of bone morphogenetic protein-9 in the regulation of glucose and lipid metabolism. *FASEB J.* **33**, 10077–10088 (2019). 31237775 10.1096/fj.201802544RR

[26] Sun, Q. J. et al. The role of bone morphogenetic protein 9 in nonalcoholic fatty liver disease in mice. *Front. Pharmacol.* **11**, 605967 (2020). 33603666 10.3389/fphar.2020.605967 PMC7884862

[27] Tang, Q. Q., Zhang, J. W. & Daniel Lane, M. Sequential gene promoter interactions by C/EBPbeta, C/EBPalpha, and PPARgamma during adipogenesis. *Biochem. Biophys. Res. Commun.* **318**, 213–218 (2004). 15110775 10.1016/j.bbrc.2004.04.017

[28] Ma, X., Wang, D., Zhao, W. & Xu, L. Deciphering the roles of PPARgamma in adipocytes via dynamic change of transcription complex. *Front. Endocrinol. (Lausanne)* **9**, 473 (2018). 30186237 10.3389/fendo.2018.00473 PMC6110914

[29] Siersbaek, R., Nielsen, R. & Mandrup, S. PPARgamma in adipocyte differentiation and metabolism–novel insights from genome-wide studies. *FEBS Lett.* **584**, 3242–3249 (2010). 20542036 10.1016/j.febslet.2010.06.010

[30] Salmon, R. M. et al. Molecular basis of ALK1-mediated signalling by BMP9/BMP10 and their prodomain-bound forms. *Nat. Commun.* **11**, 1621 (2020). 32238803 10.1038/s41467-020-15425-3 PMC7113306

[31] Al Tabosh, T. et al. Impact of heterozygous ALK1 mutations on the transcriptomic response to BMP9 and BMP10 in endothelial cells from hereditary hemorrhagic telangiectasia and pulmonary arterial hypertension donors. *Angiogenesis* **27**, 211 (2024). 38294582 10.1007/s10456-023-09902-8 PMC11021321

[32] Petrus, P. et al. Transforming growth factor-beta3 regulates adipocyte number in subcutaneous white adipose tissue. *Cell Rep.* **25**, 551-560.e5 (2018). 30332637 10.1016/j.celrep.2018.09.069

[33] Kanehisa, M., Furumichi, M., Sato, Y., Matsuura, Y. & Ishiguro-Watanabe, M. KEGG: Biological systems database as a model of the real world. *Nucleic Acids Res.* **53**, D672–D677 (2025). 39417505 10.1093/nar/gkae909 PMC11701520

[34] Kanehisa, M. & Goto, S. KEGG: Kyoto encyclopedia of genes and genomes. *Nucleic Acids Res.* **28**, 27–30 (2000). 10592173 10.1093/nar/28.1.27 PMC102409

[35] Chen, E. Y. et al. Enrichr: Interactive and collaborative HTML5 gene list enrichment analysis tool. *BMC Bioinf.* **14**, 128 (2013). 10.1186/1471-2105-14-128 PMC3637064 23586463

[36] Kasinath, V. et al. JARID2 and AEBP2 regulate PRC2 in the presence of H2AK119ub1 and other histone modifications. *Science*10.1126/science.abc3393 (2021). 33479123 10.1126/science.abc3393 PMC7993630

[37] Martinez-Hackert, E., Sundan, A. & Holien, T. Receptor binding competition: A paradigm for regulating TGF-beta family action. *Cytokine Growth Factor Rev.* **57**, 39–54 (2021). 33087301 10.1016/j.cytogfr.2020.09.003 PMC7897244

[38] Li, W. & Morrell, N. W. Endothelial bone morphogenetic protein signaling in pulmonary arterial hypertension. *Encycloped. Cell Biol.* **6**, 551–562 (2022).

[39] Aykul, S., Maust, J. & Martinez-Hackert, E. BMP-4 extraction from extracellular matrix and analysis of heparin-binding properties. *Mol. Biotechnol.* **64**, 156–170 (2022). 34550550 10.1007/s12033-021-00403-x PMC8766921

[40] Ohkawara, B., Iemura, S., ten Dijke, P. & Ueno, N. Action range of BMP is defined by its N-terminal basic amino acid core. *Curr. Biol.* **12**, 205–209 (2002). 11839272 10.1016/s0960-9822(01)00684-4

[41] Bidart, M. et al. BMP9 is produced by hepatocytes and circulates mainly in an active mature form complexed to its prodomain. *Cell. Mol. Life Sci.* **69**, 313–324 (2012). 21710321 10.1007/s00018-011-0751-1 PMC11114909

[42] Grgurevic, L., Christensen, G. L., Schulz, T. J. & Vukicevic, S. Bone morphogenetic proteins in inflammation, glucose homeostasis and adipose tissue energy metabolism. *Cytokine Growth Factor Rev.* **27**, 105–118 (2016). 26762842 10.1016/j.cytogfr.2015.12.009

[43] Huang, Z. et al. Brown adipose tissue involution associated with progressive restriction in progenitor competence. *Cell Rep* **39**, 110575 (2022). 35417710 10.1016/j.celrep.2022.110575 PMC9664906

[44] Aykul, S., Maust, J., Thamilselvan, V., Floer, M. & Martinez-Hackert, E. Smad2/3 activation regulates Smad1/5/8 signaling via a negative feedback loop to inhibit 3T3-L1 adipogenesis. *Int J Mol Sci* **22**, 8472 (2021). 34445177 10.3390/ijms22168472 PMC8395197

[45] Schreiber, I. et al. BMPs as new insulin sensitizers: Enhanced glucose uptake in mature 3T3-L1 adipocytes via PPARgamma and GLUT4 upregulation. *Sci. Rep.* **7**, 17192 (2017). 29222456 10.1038/s41598-017-17595-5 PMC5722815

[46] Schulz, T. J. et al. Brown-fat paucity due to impaired BMP signalling induces compensatory browning of white fat. *Nature* **495**, 379–383 (2013). 23485971 10.1038/nature11943 PMC3623555

[47] Long, L. et al. Selective enhancement of endothelial BMPR-II with BMP9 reverses pulmonary arterial hypertension. *Nat. Med.* **21**, 777–785 (2015). 26076038 10.1038/nm.3877 PMC4496295

[48] Yung, L. M. et al. ACTRIIA-Fc rebalances activin/GDF versus BMP signaling in pulmonary hypertension. *Sci. Transl. Med.*10.1126/scitranslmed.aaz5660 (2020). 32404506 10.1126/scitranslmed.aaz5660 PMC8259900

[49] Hoeper, M. M. et al. Phase 3 trial of sotatercept for treatment of pulmonary arterial hypertension. *N. Engl. J. Med.* **388**, 1478–1490 (2023). 36877098 10.1056/NEJMoa2213558

[50] Li, W. & Quigley, K. Bone morphogenetic protein signalling in pulmonary arterial hypertension: Revisiting the BMPRII connection. *Biochem. Soc. Trans.* **52**, 1515–1528 (2024). 38716930 10.1042/BST20231547 PMC11346422

[51] Pescador, N. et al. Metformin reduces macrophage HIF1alpha-dependent proinflammatory signaling to restore brown adipocyte function in vitro. *Redox Biol.* **48**, 102171 (2021). 34736121 10.1016/j.redox.2021.102171 PMC8577482

[52] Kim, D., Langmead, B. & Salzberg, S. L. HISAT: A fast spliced aligner with low memory requirements. *Nat. Methods* **12**, 357–360 (2015). 25751142 10.1038/nmeth.3317 PMC4655817

[53] Anders, S., Pyl, P. T. & Huber, W. HTSeq–a Python framework to work with high-throughput sequencing data. *Bioinformatics* **31**, 166–169 (2015). 25260700 10.1093/bioinformatics/btu638 PMC4287950

[54] Durinck, S., Spellman, P. T., Birney, E. & Huber, W. Mapping identifiers for the integration of genomic datasets with the R/Bioconductor package biomaRt. *Nat. Protoc.* **4**, 1184–1191 (2009). 19617889 10.1038/nprot.2009.97 PMC3159387

[55] Johnson, W. E., Li, C. & Rabinovic, A. Adjusting batch effects in microarray expression data using empirical Bayes methods. *Biostat.* **8**, 118–127 (2007). 10.1093/biostatistics/kxj037 16632515

[56] Love, M. I., Huber, W. & Anders, S. Moderated estimation of fold change and dispersion for RNA-seq data with DESeq2. *Genome Biol.* **15**, 550 (2014). 25516281 10.1186/s13059-014-0550-8 PMC4302049

[57] Sergushichev, A. A. An algorithm for fast preranked gene set enrichment analysis using cumulative statistic calculation. *BioRxiv* (2016).

[58] Alvarez, M. J. et al. Functional characterization of somatic mutations in cancer using network-based inference of protein activity. *Nat. Genet.* **48**, 838–847 (2016). 27322546 10.1038/ng.3593 PMC5040167

