# Supplementary material for: Distinct signalling dynamics of BMP4 and BMP9 in brown versus white adipocytes
Source: Sci Rep. 2025 May 8;15:15971. doi: 10.1038/s41598-025-99122-5 (PMC12059129; doi:10.1038/s41598-025-99122-5)
Supplement: Supplementary file 1 — Supplementary Information. [file 41598_2025_99122_MOESM1_ESM.pdf]

Distinct Signaling Dynamics of BMP4 and BMP9 in Brown versus White Adipocytes

Benjamin Constant[§,1], Ioannis Kamzolas[§,2,3], Xudong Yang[1], Jingxu Guo[1], Sonia Rodriguez-Fdez[1,2], Iman Mali[1,2], Sergio Rodriguez-Cuenca[2], Evangelia Petsalaki[3], Antonio Vidal-Puig[$,1,2,*], Wei Li[$,1,*]

Supplementary Figure 1. **Time course signalling assays**.

Supplementary Figure 2. **A summary of the GSEA analysis of BMP4 and BMP9 regulated DEGs in white adipocytes.**

Supplementary Figure 3. **EnrichR analysis of DEGs from BMP4- and BMP9- treated brown and white adipocytes.**

Supplementary Figure 4. **In brown adipocytes, BMP4 and BMP9 do not induce significant changes in the expression of TGF-β family receptor.**

Supplementary Figure 5**. Uncropped blot images for Supplementary Figure 1.**

Supplementary Table 1. **Primers for the RT-qPCR analysis.**

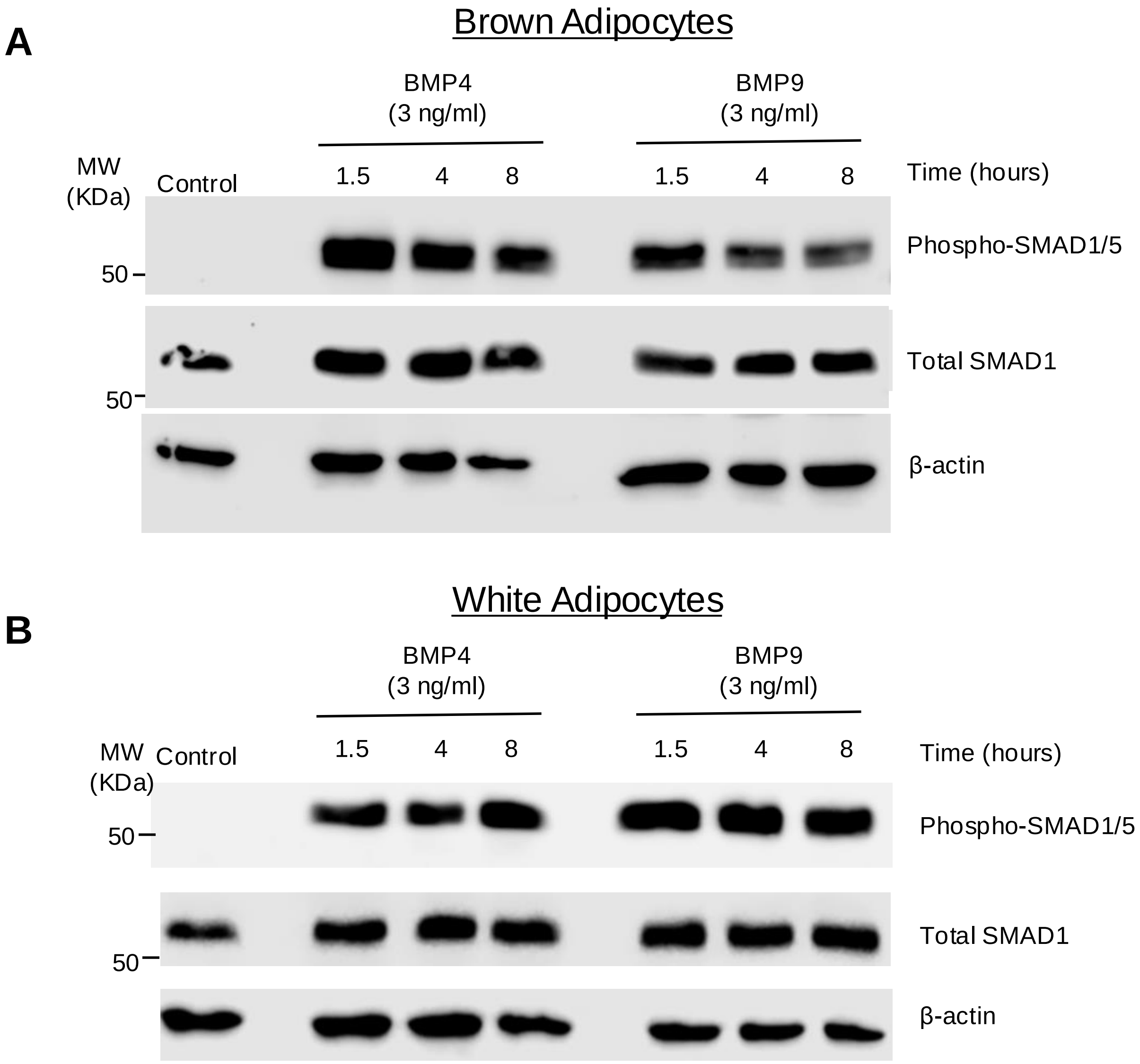

Supplementary Figure 1. Time course signalling assays. Mature brown and white adipocytes were treated with BMP4 or BMP9 for the indicated length of time, and Smad1/5 phosphorylation were measured by immunoblotting. An identical gel was run for total Smad1 immunoblot. β-actin was used as a loading control. Original blots/gels are presented in Supplementary Figure 5.

		**Gene Ontology (GO)**	**KEGG**	**Reactome**
BMP4	Up-regulated	165	39	28
	Down- regulated	19	10	2
BMP9	Up-regulated	98	15	69
	Down-regulated	8	5	4

Supplementary Figure 2. A summary of the GSEA analysis of BMP4 and BMP9 regulated DEGs in white adipocytes. Number of up- and down-regulated terms with adjusted P-values smaller than 0.05.

A

	Number of terms with AdjP <0.05	GO Biological process	GO Cellular component	GO Molecular function	KEGG
1	BMP4, in both brown and white adipocytes	**67**	**none**	**16**	**3**
2	BMP9, in both brown and white adipocytes	**85**	**none**	**14**	**3**
3	BMP4, only in white adipocytes	**139**	**4**	**2**	**25**
4	BMP4, only in brown adipocytes	**none**	**1**	**none**	**1**
5	BMP9, only in white adipocytes	**93**	**1**	**8**	**24**
6	BMP9, only brown adipocytes	**4**	**none**	**none**	**1**
7	White adipocytes, common in both BMPs	**177**	**1**	**17**	**23**
8	White adipocytes, only BMP4	**5**	2	**none**	**1**
9	White adipocytes, only BMP9	**none**	**none**	**none**	**none**
10	Brown adipocytes, common in both BMPs	**88**	**1**	**13**	**3**
11	Brown adipocytes, only BMP4	**none**	**none**	**none**	**none**
12	Brown adipocytes, only BMP9	**12**	**none**	**none**	**2**

B

White adipocytes, only BMP4 (row 8)

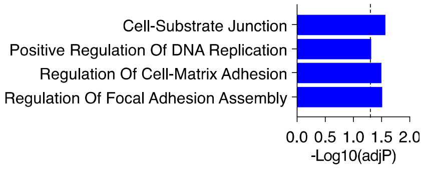

C

Brown adipocytes, only BMP9 (row 12)

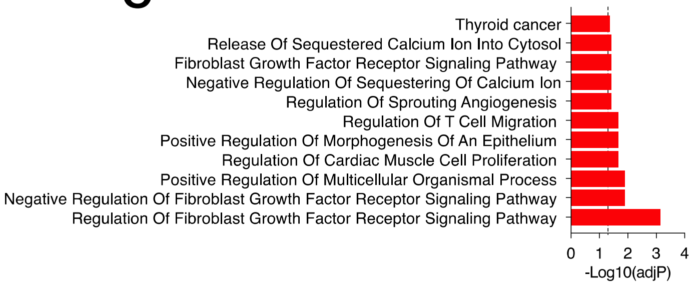

Supplementary Figure 3. EnrichR analysis of DEGs from BMP4- and BMP9-treated brown and white adipocytes. A. A summary of total number enriched terms with adjusted P-values smaller than 0.05. **B**. In white adipocytes, significantly enriched pathways can be found for only BMP4 treatment. **C**. In brown adipocytes, significantly enriched pathways can be found only BMP9 treatment.

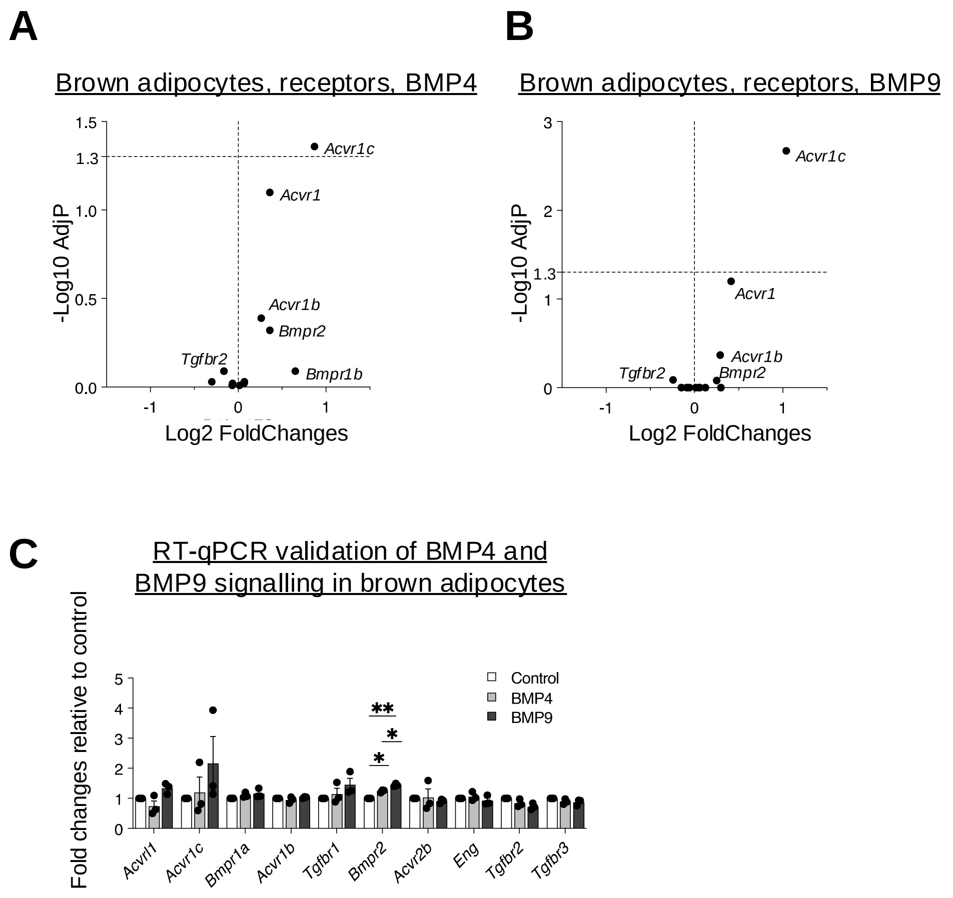

Supplementary Figure 4. In brown adipocytes, BMP4 and BMP9 do not induce significant changes in the expression of TGF-β family receptor. A&B. Volcano plots of differential gene expressions of TGF-β family receptors. A dashed line is drawn at Y=1.3 which indicate where the adjP =0.05. Only induction of Acvr1c has adjP <0.05. **C**. RT-qPCR validation of receptors changes using three independently differentiated brown and white adipocytes, treated with control or BMPs. Means ± SEM are shown. Two-way ANOVA. *, P<0.05, **, P<0.01.

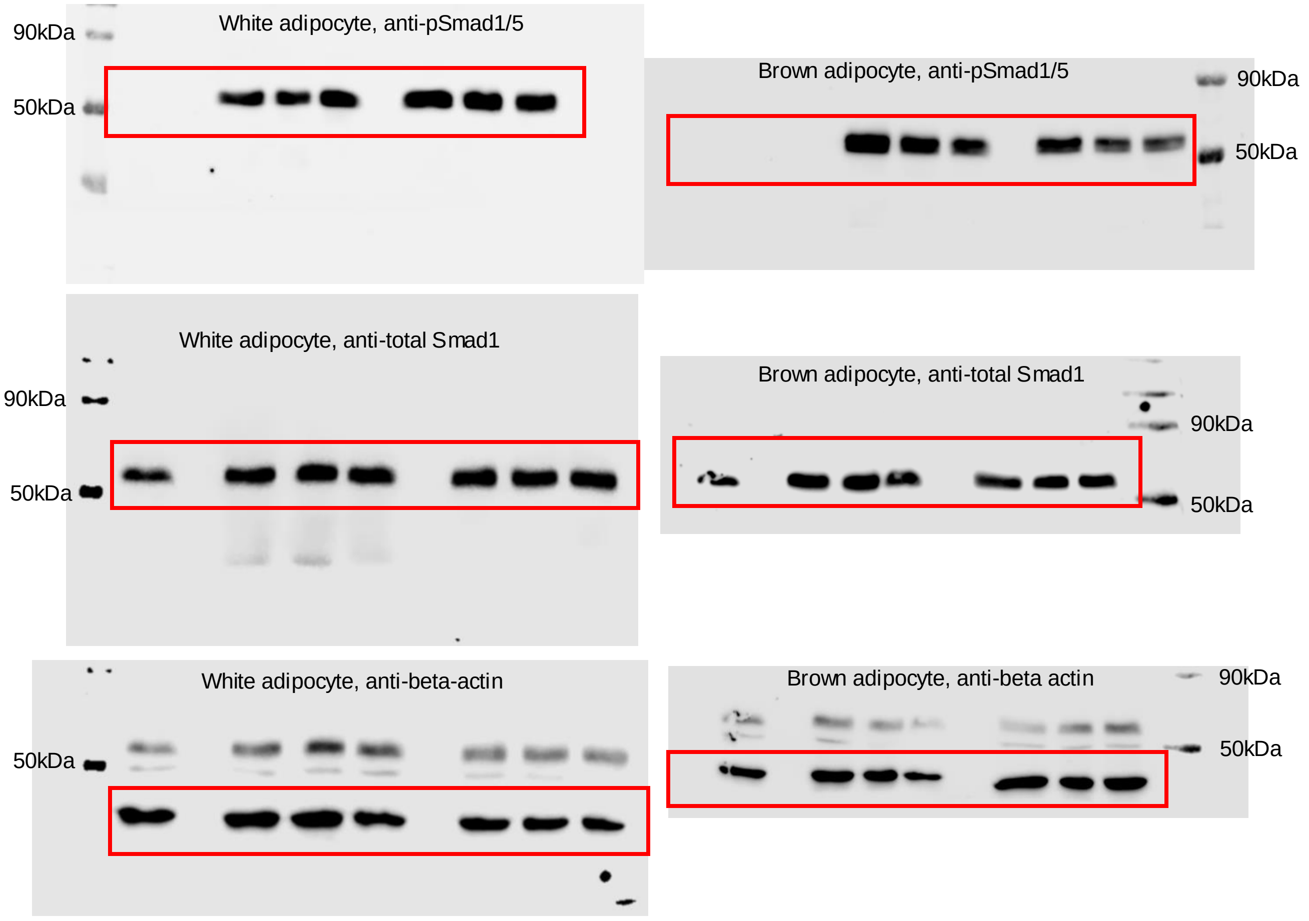

Supplementary Figure 5. Uncropped blot images for Supplementary Figure 1.

Supplementary Table 1. Primers for the RT-qPCR analysis.

Gene	Primer F	Primer R
Actb	GGGAAATCGTGCGTGACAT	GTGATGACCTGGCCGTCAG
Acvr1	QuantiTect Primer Assay	GeneGlobe ID:QT00093422
Acvr1b	QuantiTect Primer Assay	GeneGlobe ID:QT00163464
Acvr1c	QuantiTect Primer Assay	GeneGlobe ID: QT00262591
Acvr2a	QuantiTect Primer Assay	GeneGlobe ID: QT00147602
Acvr2b	QuantiTect Primer Assay	GeneGlobe ID: QT00109039
Acvrl1	QuantiTect Primer Assay	GeneGlobe ID: QT00161434
Adipoq	GAGAGAAAGGAGATGCAGGTCTTC	ACACATAAGCGGCTTCTCCAG
Apln	AGGCATAGCGTCCTCACCTCTT	GGTGCAGAAACGACAAAGACGG
B2m	GTATACTCACGCCACCCACC	TGGGGGTGAATTCAGTGTGAG
Bmp4	GCCGAGCCAACACTGTGAGGA	GATGCTGCTGAGGTTGAAGAGG
Bmpr1a	QuantiTect Primer Assay	GeneGlobe ID:QT01057511
Bmpr1b	QuantiTect Primer Assay	GeneGlobe ID: QT00121240
Bmpr2	QuantiTect Primer Assay	GeneGlobe ID: QT00251349
Ccn1	GTGAAGTGCGTCCTTGTGGACA	CTTGACACTGGAGCATCCTGCA
Ccn2	TGCGAAGCTGACCTGGAGGAAA	CCGCAGAACTTAGCCCTGTATG
Cebpa	TTGCACCTCCACCTACATCC	CCACAAAGCCCAGAAACCTA
Elovl7	TCAGTCGCCAAGAGCAATGAGG	GACATGAAGGAAAGTCACTTGGC
Eng	CAGCCAAAGTGTGGCAATCAGG	GCTACTCAGGACAAGATGGTCG
Fabp4	TTCCTTCAAACTGGGCGTGG	CGCCTTTCATAACACATTCCACC
Fasn	TTGGCCTACACCCAGAGCTA	GTGATAAGGTCCACGGAGGC
Foxp2	GCATTGGATGACCGAAGCACTG	TCGCATGTGCAAGTGGGTCATC
Glut4	ATTGCAGCGCCTGAGTCTTT	GGTTCCCCATCGTCAGAGC
Hif1a	CCTGCACTGAATCAAGAGGTTGC	CCATCAGAAGGACTTGCTGGCT
Hprt	CTTCCTCCTCAGACCGCTTT	ATCGCTAATCACGACGCTGG
Id1	GCTCACTTTGCGGTTCTGG	ACGACATGAACGGCTGCTACT
Jarid2	GCCTGCATAAAGAAGCACCTCAG	GAGGTCAGTCACTTGCTGCATG
Lipe	AGAAGGATCGAAGAACCGCAG	GTGTGAGAACGCTGAGGCTTTG
Pgc1a	ATGGAGTGACATAGAGTGTGCTG	TTCCGATTGGTCGCTACACC
Plin1	AGTGAGTGTGGGGTCCTTGG	GACATCCTTACTCTCCACGCTGT
Pparγ	GCCCTTTGGTGACTTTATGG	CAGCAGGTTGTCTTGGATGT
Rassf4	ACTGAAGAGCCTGGCACTCAAC	CATCGCTCTTTGTCCGCATAAGC
Syngr3	ACGTGCGCTTTCAGCAGATCAG	TCGTTGCCACTGATTGGTGAGG
Tead1	TCCGCTTTCCTTGAACAGCAGAG	GGGTCACTGTAAGAATGGTTGGC
Tgfβr1	QuantiTect Primer Assay	GeneGlobe ID: QT00135828
Tgfβr2	QuantiTect Primer Assay	GeneGlobe ID: QT00135646
Tgfβr3	GGTGTGAACTGTCACCGATCA	GTTTAGGATGTGAACCTCCCTTG
